# Biomimetic core-shell GelMA microspheres co-delivering ANXA1, NGF, and fibronectin enable phase-matched immunomodulation and neurorepair after spinal cord injury

**DOI:** 10.7150/thno.120426

**Published:** 2026-01-01

**Authors:** Youjun Liu, Chunping Hu, Siyuan He, Renfeng Liu, Yuqi Zhao, Yuhao Wang, Hailiang Xu, Hui Li, Yanming Ma, Botao Lu, Yixiang Ai, Cheng Ju, Weidong Wu, Yifan Wang, Dageng Huang, Dingjun Hao, Zhiyuan Wang, Baorong He, Lei Zhu

**Affiliations:** 1 Department of Spine Surgery, Honghui Hospital, Xi'an Jiaotong University, Xi'an, Shaanxi 710054, China.; 2 Shaanxi Key Laboratory of Spine Bionic Treatment, Xi'an, Shaanxi 710054, China.

**Keywords:** Spinal cord injury, GelMA microsphere, Anti-inflammatory, Nerve regeneration, Spatiotemporal delivery

## Abstract

**Background:** Spinal cord injury (SCI) leads to permanent sensory and motor function loss, characterized by inflammation and neuronal loss. A promising therapeutic strategy involves delivering anti-inflammatory and neuroregenerative agents tailored to these phases.

**Methods:** GelMA-AFN hydrogel microspheres were prepared by a UV-crosslinked microfluidic chip. Immunofluorescence was performed to assess the effect of GelMA-AFN on apoptosis, axonal growth in dorsal root ganglion (DRG) neurons. Immunohistochemistry, flow cytometry, electrophysiology, RNA-seq, and behavioral testing were used to evaluate histological and functional recovery in a rat SCI model.

**Results:** In this study, we developed GelMA-AFN with a dual-layer structure and an mean diameter of 50 µm. The outer layer, containing low-concentration gelatin methacryloyl (GelMA, 5%) and annexin A1 (ANXA1), provided sustained ANXA1 released for up to 7 days, while the inner layer, with high-concentration GelMA (10%), nanoclay, fibronectin (FN), and nerve growth factor (NGF), releases FN and NGF over 6 weeks. *In vitro*, GelMA-AFN inhibits neuronal apoptosis, promoted axonal growth, and enhances survival under oxidative stress. *In vivo*, it reduced early inflammation by limiting neutrophil recruitment and promoting macrophage M2 polarization. Eight weeks post-SCI in a rat model, GelMA-AFN enhanced axonal extension, myelin regeneration, beneficial ECM deposition, and reduced glial scar formation, leading to significant neural electrical signal conduction and motor function recovery. mRNA-seq analysis confirmed GelMA-AFN upregulates genes associated with anti-inflammatory responses and axonal extension while downregulating pro-inflammatory genes.

**Conclusion:** These results suggest GelMA-AFN as a promising therapeutic approach for SCI by providing spatiotemporal delivery aligned with the injury's dynamic stages.

## Introduction

Spinal cord injury (SCI) arises from trauma, tumors, or inflammation and frequently causes axonal damage and neuronal death, leading to deficits in movement, sensation, and autonomic function 1. Annually, over a million new SCI cases are reported worldwide, with an estimated 27 million people living with varying degrees of post-SCI symptoms 2. In China, there are more than one million SCI patients, with the number growing by approximately 120,000 each year 3. SCI markedly reduces quality of life and imposes a substantial burden on healthcare systems 4. Unfortunately, no effective disease-modifying therapies for SCI are available in clinical practice. SCI pathophysiology comprises primary and secondary injury phases. Primary injury, caused directly by trauma, leads to the destruction of neural tissue and necrosis of neurons and glial cells. Secondary injury, including oxidative stress, delayed apoptosis, demyelination of peripheral neurons, and inflammatory responses, exacerbates tissue damage and neurological deficits 5. Secondary injury triggers influxes of neutrophils, macrophages, T cells, and microglia and the release of numerous cytokines, thereby disrupting the blood-spinal cord barrier and initiating inflammatory cascades that cause additional tissue damage. This exacerbated neuronal loss and tissue damage impair synaptic remodeling and neural circuit regeneration, becoming a major obstacle to SCI repair 6. Additionally, inflammatory cytokines and vascular disruption after SCI often reduce neurotrophic support, further hindering regeneration. Inflammatory cells not only clear necrotic tissue but also disrupt the spinal cord extracellular matrix (ECM). ECM components such as fibronectin and laminin provide a scaffold that supports neuronal adhesion and extension. The ECM in the spinal cord aids in guiding axonal growth and synapse formation during neural development and regeneration, and its disruption has adverse effects on SCI recovery 7. Neuroinflammation is a key component of secondary injury that diminishes neurotrophic signaling and degrades ECM structure, thereby impeding recovery 8. It is believed that the inflammatory environment significantly improves 3-4 weeks after SCI, at which point the repair of damaged neurons begins 9. However, the innate capacity for repair is limited and insufficient to restore function fully. Thus, developing composite materials that combine anti-inflammatory, neurotrophic, and ECM-supporting functions represents a promising direction for SCI treatment 10.

Modulating the post-SCI inflammatory microenvironment has long relied on steroids and nonsteroidal anti-inflammatory drugs (NSAIDs) 11. However, high dosages are associated with significant side effects. Annexin A1 (ANXA1) is an anti-inflammatory protein that mediates glucocorticoid responses and regulates innate immune processes. ANXA1 exhibits potent anti-inflammatory activity, mediating glucocorticoid's anti-inflammatory effects through G-protein-coupled formyl peptide receptor 2 (FPR2/ALX). ANXA1 can inhibit neutrophil migration, promote neutrophil apoptosis, suppress astrocyte release of pro-inflammatory mediators, inhibit activation of macrophages/microglia, and encourage polarization of macrophages/microglia towards an M2 phenotype 12-14. In circulating granulocytes and monocytes, ANXA1 is abundant and primarily cytoplasmic under resting conditions. Upon inflammatory stimulation and neutrophil adhesion to endothelium in postcapillary venules, granule exocytosis drives ANXA1 translocation to the cell surface, where it anchors in a calcium-dependent manner. Surface-anchored ANXA1 interacts with adhesion molecules that mediate leukocyte-endothelial cell interactions, thereby inhibiting leukocyte migration to the site of inflammation 15, 16. ANXA1 also contributes to cytoskeletal and ECM integrity, is expressed in most human organs, and exhibits minimal immunogenicity 17-19. Exogenously administered ANXA1 plays a crucial role in treating various diseases, including myocardial infarction, cerebral ischemia, and rheumatoid arthritis 20-22. However, applications of ANXA1 in SCI remain limited, and its potent anti-inflammatory properties are underexplored. This study proposes the early release of ANXA1 protein to inhibit the inflammatory response in the early stage of SCI, improving the microenvironment.

Neurotrophic factors are crucial for neural development, repair, and regeneration. Research by Sofroniew MV *et al.* has shown that after SCI, both astrocytes and non-astrocytes in the spinal tissue nearly cease to express GDNF, BDNF, NT3, and other neurotrophic factors 23. Therefore, supplementing appropriate exogenous neurotrophic factors is crucial for SCI repair. Nerve growth factor (NGF) enhances cellular nutrient uptake and protein synthesis, thereby supporting neural repair. NGF binds TrkA receptors on neurons to promote survival 24. Furthermore, NGF can reduce demyelination, promote recovery from injury, and support nerve regeneration, guiding the directional growth of axons 25, 26. However, axonal growth requires appropriate adhesive substrates, which are scarce in the injured cord. The ECM acts as a scaffold in cell growth, regulating cell survival, proliferation, morphogenesis, and differentiation, among other functions. Additionally, the ECM can modulate signal transduction activated by various bioactive molecules such as growth factors and cytokines 27. Within spinal tissue, ECM components can partially counteract injury-induced inhibitory cues 28. Fibronectin (FN), a key ECM component, regulates cell adhesion and growth and supports durable cell-cell and cell-matrix interactions that preserve morphology 29, 30. Accordingly, FN is well suited as an exogenous neuro-supportive substrate to promote axonal adhesion, extension, and regeneration. However, co-integrating ANXA1, NGF, and FN into a single slow-release platform with temporally and spatially controlled delivery remains challenging.

With advances in tissue engineering, combining biomaterials with therapeutics offers new approach for SCI repair. Hydrogels, characterized by their ECM-like water content, thixotropy, and ideal biocompatibility, can serve as "carriers" to load various elements beneficial for neural growth and microenvironment improvement, such as cells, drugs, and proteins 30. Hydrogels have been widely applied in basic research on SCI treatment. A key challenge is integrating early anti-inflammatory action with sustained neurotrophic delivery within a single hydrogel system. Methacrylated gelatin (GelMA) is promising for scaffolds and drug delivery owing to its biocompatibility and tunable mechanics 31. GelMA contains a high number of arginine-glycine-aspartic acid sequences and matrix metalloproteinase-sensitive degradable sequences, similar to natural ECM, making it suitable for cell binding and adhesion 32. Additionally, GelMA can form crosslinked networks of hydrogels through photo-polymerization under mild conditions, offering temporal and spatial control, different structures, flexibility, softness, superior colloidal stability, and a large surface area allowing multivalent bioconjugation, considered a forward-looking scaffold for microtissue engineering 33. Traditional GelMA hydrogels degrade within approximately 14 days *in vivo*, an insufficient time frame for effective repair and regeneration amidst the surrounding inflammatory environment 34. We therefore incorporated nanoclay—an inorganic nano-additive—into GelMA to enhance mechanical performance, adhesion, and printability 35, 36. Nanoclay incorporation also improves cell-scaffold interactions, supporting cell proliferation and stem-cell differentiation 37. Nano-clay enhanced GelMA hydrogels degrade over a month in rats, matching the onset of neural repair after SCI 38. Bulk hydrogels are difficult to inject, contact tissue unevenly, and can release cargo nonuniformly; microspheres can mitigate these issues 39. Traditional microsphere preparation methods like physical rupture, emulsion, and electrospray have drawbacks such as unstable microsphere size and shape, and low yield. Microfluidic synthesis enables high-quality microspheres with uniform size and morphology 40. Therefore, we employed microfluidic technology to prepare a large quantity of dual-layer, multifunctional GelMA hydrogel microspheres (GelMA-AFN), with the outer layer of low-concentration GelMA encapsulating ANXA1 and the inner layer of high-concentration GelMA mixed with nano-clay encapsulating NGF and FN, achieving complete degradation over a month or more.

In this study, we fabricated temporally sequenced, multifactorial slow-release GelMA microspheres by microfluidics. Through a dual-encapsulation microsphere chip, ANXA1 was encapsulated in the outer low-concentration GelMA layer, while NGF and FN were encapsulated in the inner high-concentration GelMA layer mixed with nano-clay. The outer layer achieves early-stage release, inhibiting inflammatory responses, while the inner layer sustains release for over a month, continuously releasing neurotrophic factors and specific ECM, promoting axonal regeneration. This treatment strategy mimics the dynamic pathological changes after SCI. *In vitro* experiments used fluorescence staining to identify the structure of the prepared microspheres, validating their compatibility with nerve cells and their functionality in improving inflammation and promoting neural repair. Finally, we tested these microspheres in a rat SCI model and evaluated inflammation, neuronal apoptosis, axonal growth and remyelination, and motor recovery by histopathology and behavioral assays.

## Materials & Methods

### Fabrication of GelMA-AFN

The core of the microspheres was prepared using GelMA (EFL, China), nanoclay (BYK, UK), NGF (Beyotime, China), FN (Beyotime, China), and LAP (EFL, China). Nanoclay powder was dispersed in deionized water, the compound was stirred continuously overnight at indoor temperature, resulting in an entirely soluble solution with a concentration of 3 wt%. A 20 wt% GelMA solution, with a 90% amino substitution ratio, was prepared by dissolving GelMA in deionized water and heating it in a 60 °C bain-marie for 30 min. The photoinitiator LAP (0.05 g/mL) was added and mixed dissolve fully. The hydrogel solution was then mixed with a 1:1 ratio of the nanoclay solution, then placed in a lucifugal incubator at indoor temperature for 2 h. The obtained compound containing 10 wt% GelMA and 1.5 wt% nanoclay. NGF was introduced to reach a concentration of 100 μg/mL, while FN was added to achieve a concentration of 10 μg/mL. The shell of the microspheres was composed of GelMA, ANXA1 (Abcam, UK), and LAP. Solid GelMA with 30% amino substitution was stirred with deionized water, 0.01 wt% Span 80 (Sinopharm, China), and heated in a 60 °C water bath for 30 min, producing 5 wt% GelMA hydrogel solution. After the solid GelMA was fully dissolved, LAP was added. After the mixture cooled down, ANXA1 protein was added to the solution to reach a content of 100 μg/mL. The oil phase consists of dimethyl silicone oil (Dow Corning, USA) with a viscosity of 100 cSt.

To generate the microspheres, the aforementioned solutions were injected into the microfluidic chip. At the ideal flow ratio, optical microscopy identified many microdroplets exhibiting a unique core-shell configuration. These microdroplets displayed consistent sizes and a stable output. A UV source (EFL, China, 58.8 mW/cm^2^, 365 nm, 20 s) was employed at the S-shaped channel to facilitate the crosslinking of the GelMA microdroplets, resulting in the creation of core-shell microgels. The microspheres flow into the collecting pool through the tubing, thereby allowing for the collection of a substantial quantity of GelMA-AFN with a diameter of 50 μm. After collecting sufficient pellets, most dimethyl silicone oil was eliminated through differential centrifugation at 1200 rpm for 3 min. The microspheres were then immersed in PBS for 3 min, repeating this process three times, and finally stored at 4 °C.

### Characterization of GelMA-AFN

Morphology of microspheres: Diameters at different flow ratios were imaged on a THUNDER Imager Tissue microscope (Leica, Germany) and quantified in ImageJ.

Structure of microspheres: The presence of the core-shell architecture was verified through fluorescent staining techniques. To synthesize the fluorescent microspheres, rhodamine fluorescent dye (Merck, Germany) was incorporated into the shell solution, excluding the core solution. The outcomes of the staining were subsequently examined under a THUNDER Imager Tissue fluorescence microscope (Leica, Germany).

Degradation of the microspheres *in vitro*: Fluorescent microspheres were created by integrating an appropriate quantity of rhodamine fluorescent dye (Merck, Germany) into the outer layer's solution, with the inner layer's solution remaining dye-free or fluorescein isothiocyanate (FITC, Merck, Germany). Subsequently, GelMA-AFN bilayer microspheres with a diameter of 50 μm were soaked in PBS at 37 °C. The fluorescence staining changes were observed at particular points in time (days 1, 3, 5, 7, 9) using a fluorescence microscope. This assay examined the degradation of the outer sphere. After the complete degradation of the outer sphere, sufficient pellets of the inner microsphere were immersed in PBS at 37 °C to assess their degradation properties. At days 7, 14, 21, 28, 35, and 42, supernatant was removed and samples were weighed.

Degradation of the microspheres *in vivo*: During the preparation of GelMA-AFN, rhodamine dye was incorporated into the outer layer of the microsphere, while FITC was introduced into the inner layer. Subsequently, the microsphere was injected into the center of the spinal cord injury lesion area of SCI rats using a microinjector. Tissue samples from the center of the spinal lesion were collected on days 1, 3, 5, 7, 14, and 28 post operation, and the fluorescence intensity of both dyes was quantitatively assessed using fluorescence microscopy (DMi8 Thunder, Leica) and ImageJ 1.51.

Swelling ratio: Full samples of GelMA-AFN pellets and equally sized bilayer GelMA pellets, which did not contain nanoclay, were dipped in PBS at 37 °C and incubated for 24 h. After removing surface moisture, the swelling of the samples was assessed, and their weights were recorded at various time points (0, 4, 8, 12, 16, 20, and 24 h).

Drug encapsulation efficiency and drug loading capacity: GelMA-AFN was immersed in a PBS solution. After the complete degradation of the outer layer, the supernatant was collected by centrifugation, and the encapsulation efficiency and drug loading capacity of ANXA1 was determined using high-performance liquid chromatography. After heating of the precipitate, NGF and FN were separated using an adsorption column, and their respective encapsulation rates and drug loading capacity were calculated.

Drug release: GelMA-AFN pellets (100 μL) were immersed in 1 mL of PBS at 37 °C, and the corresponding proteins were detected using ELISA kits (Beyotime, China). The concentrations of ANXA 1 protein (Proteintech, USA) were measured on days 1, 3, 5, and 7, while the cumulative release ratios of NGF and FN (Beyotime, China) were determined on days 1, 7, 14, 21, 28, and 35.

### DRG culture

Newborn Sprague-Dawley rats were sterilized by immersion in 75% ethanol. Subsequently, the dorsal root ganglia (DRGs) were carefully excised using Sterile microscope tweezers. Following this, the excised tissue was rinsed with chilled D-PBS solution and segmented into bits of tissue suitable for explant culture. Cell suspensions were prepared by mechanically disrupting the DRG tissue blocks and digesting them in a solution containing 0.25% trypsin and collagenase type IV for 30 min. The resultant homogenate was sequentially filtered through 40 μm cell filter. Both DRG neurons and explants were placed into culture dishes. The DRG neurons were dilute to a concentration of 1 × 10^6^/mL, and then seeded into culture dish containing Neurobasal medium which including 2% B27 and 1% glutamine. Additionally, the DRG explants based on the experimental purpose were cultured respectively in the different culture dish with conditioned medium. On day 7, neurons or explants were labeled with Tuj1. The GelMA-AFN microspheres co-cultured with DRG explants were pre-immersed in sterile PBS for over seven days, resulting in the complete degradation of their outer layer. Images of various sights were randomly taken with a microscope (DMi8 Thunder, Leica). ImageJ software was used to evaluate the growth of nerve extensions, based on the the total area of nerve protrusions and mean length of the 50 longest axons 41.

### Cell apoptosis assay

To assess the impact of GelMA-AFN on the apoptosis of DRG neurons, DRG neurons were incubated in various media for 24 h, following previously established protocols. Subsequently, a TUNEL assay was test by a One-step TUNEL Apoptosis Kit (red, AF488, C1170, Beyotime, China). Using 4% paraformaldehyde to fix cells, and then permeabilizing cells in a 0.3% Triton X-100 solution for 15 min. After a 30-minute incubation with TdT buffer at 37 °C, the cells received the labeling solution for 60 min in a light-sheltered environment and subsequently stained with DAPI. The fluorescence microscope (THUNDER Imager Tissue) was used to capture images, and using ImageJ software to quantifiy apoptotic cells, with observers remaining blinded to the experimental conditions. The ratio of apoptotic cells to the total number of cells was calculated.

### Live/dead assay

Cells were co-cultured with microspheres for a duration of 24 h. Following guidelines, the ratio of dead or live cell was assessed using the Calcein/PI Cell Viability/Cytotoxicity Assay Kit (Beyotime, C2015S, China). Fluorescence images were captured using a microscope (DMi8 Thunder, Leica), and cell viability was counted using the formula: cell viability (%) = (the number of Calcein-AM^+^ cells) / (the number of Calcein-AM^+^ cells + the number of PI^+^ cells) × 100%. The ratio of live/dead cells to total cells is used as an indicator of survival/death.

### Cellular activity in inflammatory environment

After 3 days of culturing neurons, three groups of cells were exposed to a 200 μmol/L hydrogen peroxide solution for 30 min. The medium was then replaced, and the culture dishes were processed according to group-specific protocols, followed by an incubation period of 24 h. The proportion of dead to living cells was quantified using a Cell Death Kit (Beyotime, C2015M, China). Random images were observed using a high-precision fluorescence microscope (DMi8 Thunder, Leica), and the number of living cells was accurately estimated using ImageJ software.

### SCI model and transplantation surgeries

Adult female Sprague-Dawley (SD) rats weighing between 220-230 g (*n* = 96) were purchased from the Laboratory Animal Center of Xi'an Jiaotong University (Shannxi, China) for the SCI model. All animal-related procedures were performed in compliance with the ethical guidelines set forth by the Animal Care and Use Committee of Xi'an Jiaotong University (XJTUAE2024-1572). Rats were anesthetized by intraperitoneal injection of 1% sodium pentobarbital at a dose of 40 mg/kg. The fur on their backs was shaved and cleaned using iodophor (310102, Shanghai Likang Disinfection Hi-Tech Co, China). The T10 was located based on the highest point of the rat's prone position, and a partial laminectomy was operated around the T8 vertebra for exposing the T8 spinal cord. Then, we clamped the spinal cord for 30 s using micro-pliers 42 (Figure S1A).

The rats subjected to T8 injury were randomly distributed to four groups (Animals were numbered sequentially and assigned to groups based on SPSS-generated random numbers, ensuring each animal had an equal opportunity for group assignment, which helps to minimize potential biases): microinjection of GelMA-AFN (GelMA-AFN, 10 μL, *n* = 24), microinjection of core-shell GelMA microspheres without protein (GelMA, 10 μL, *n* = 24), spinal cord injury (SCI, *n* = 24), and sham-operated (Sham, *n* = 24). In each 10 μL injection, 9 μL comprised purified microspheres and 1 μL PBS to improve injectability. The injection site was the medianus posterior sulcus of T8 spinal cord, and the injection depth was 1mm (Figure S1A). microinjections were conducted one day post-spinal cord injury with the aid of a digital stereotactic injector (51709, Stoelting Co. USA), the flow rate was 1 μL/min. To guarantee adequate tissue infiltration, the injector tip remained stationary for an extra 5 min following each injection. After transplantation, the overlying musculature and skin were sutured using 6-0 nylon sutures (Ethicon, San Lorenzo, Puerto Rico, 667G). The bladders of the rats were manually expressed three times daily until spontaneous urination resumed. Subsequent histological, functional, and behavioral assessments of the animals were performed by experimenters who were unaware of the group assignments.

### Flow cytometric analysis

Rats of spinal cord injury group (SCI, *n* = 6), core-shell GelMA microspheres without protein transplantation group (GelMA, *n* = 6), and GelMA-AFN transplantation group (GelMA-AFN, *n* = 6), and the sham-operated group (sham, *n* = 6) were used for flow cytometry. Seven days post-surgery, a 5-mm segment from the injury site was collected for flow cytometric. The spinal cord tissues were fragmented into small pieces and filtered through 200-mesh nylon nets. After undergoing centrifugation at 4 °C and 1200 rpm for 5 min, the suspended cells were incubated with specific antibodies at 4 °C for 1 h and subsequently fixed with 1% paraformaldehyde. In this process, the antibodies were mouse anti-CD11b-APC (1:20, 562102, BD Pharmingen), rat anti-CD45-PE (1:50, 202207, Biolegend), mouse anti-CD206-PE (1:50, 141717, Biolegend), and mouse anti-CD86-FITC (1:20, 561961, BD Pharmingen). Analysis of cells was carried out using a BD Biosciences flow cytometer (USA), with subsequent data processing conducted in FlowJo 7.6 software. CD11b^+^ myeloid cells were first identified. Macrophages and microglia were then distinguished based on CD45 expression, with CD45^high^ indicating macrophages and CD45^low^ indicating microglia. M1 macrophages and microglia were identified using CD86 labeling, whereas M2 macrophages and microglia were marked with CD206 43.

### Spinal cord electrophysiology

Eight weeks post-surgery, animals from each group were anesthetized using a 40 mg/kg pentobarbital sodium solution and fixed in a stereotaxic apparatus. Motor evoked potentials (MEPs) were measured using the NeuroExam M-800 Data Acquisition Analysis System (MEDCOM, Zhuhai, China). Briefly, a bipolar stimulation electrode consisting of two tungsten microelectrodes, each measuring 85 mm (0.5 MOhm, KeDouBC) was separated by a distance of 1 mm and inserted 300-500 μm into the spinal cord's T6 segment. Single square wave pulses of 0.1 ms duration were delivered at intervals of 3 s. Electrical signals were captured using a silver sphere electrode, 1 mm in diameter, situated at the T10 segment.

### Behavioral assessments

After surgery, once the rats had acclimatized to the environment, they were able to explore the open space freely for for a duration of 3 min. The Basso, Beattie, and Bresnahan (BBB) scale were applied to estimate behavioral function of rats once a week for 8 weeks, including theforelimb/hindlimb coordination, frequency and quality of hindlimb movements. The range of BBB scores was from 0 (flaccid paralysis) to 21 (normal gait).

8 weeks after injury, the GREEN Walk system, along with its associated software (China), is utilized to accurately evaluate the coordination, footprint, and basal motor behavior, during successive move along a designated pathway. Generally, this system, comprising a narrow hallway featuring a glass panel lit by green LEDs and a high-speed camera positioned beneath was employed to evaluate functional recovery in rats. Rats was permitted to traverse the pathway while consecutive runs were captured from beneath the glass using a digital camera for a footprint assessment. In this experiment, A series of were examined: 1) maximum area, which indicates the largest surface area of the paw making contact with the glass plate during a single step; 2) step length, which measures the distance between consecutive placements of the same claw; 3) base of support, defined as the average width between the hind paw footprints.

### Muscle atrophy analysis

Eight weeks after SCI, the gastrocnemius muscle was carefully separated and weighed prior fixation. All six segments of the gastrocnemius muscle from each rat were chosen for hematoxylin and eosin (H&E) staining. Under a bright-field microscope (Leica DMIRB), images of random fields from each muscle segment were taken at a standard magnification of 200x. The cross-sectional area of muscle fibers was analyzed using ImageJ software.

### Perfusion, sectioning, and immunofluorescence staining

Perfusion and sectioning procedures were carried out at 3 days and 8 weeks post-SCI, consistent with previously established methods. Rats were deeply anesthetized with a 1% sodium pentobarbital at a dose of 40 mg/kg, followed by transcardial perfusion with ice-cold heparinized saline (10 U/mL) and subsequent fixation with a 4% paraformaldehyde (PFA) solution. The spinal cord underwent dissection and was preserved in a 4% PFA solution for 48 h at 4 °C, and subsequently underwent gradient dehydration with a sucrose solution. A segment of spinal cord tissue measuring 8-10 mm and centered on the lesion was cryopreserved and then sliced into 16 μm sections. The spinal cord sections underwent a washing process in PBS for 15 min and then were permeabilized using a 0.1% Triton-X100 for a duration of 10 min. This was followed by treatment with a 5% BSA for 40 min. After that sections were incubated with antibodies, including anti-Tuj1 (1:500; Abcam), anti-GFAP (1:300; Abcam), anti-Syn (1:400; Santa Cruz), anti-NF (1:500; Abcam), anti-MBP (1:400; Proteintech), anti-FN (1:500; Abcam), anti-LN (1:500; Abcam), anti-MPO (1:200; Proteintech), anti-IBA-1 (1:200; Abcam), anti-IL-10 (1:300; Abcam), anti-IL-1β (1:200; Abcam), anti-Caspase-3 (1:200; Abcam), anti-Neun (1:200; Abcam), anti-FPR2 (1:100; Abcam), anti-Col1 (1:200; Abcam), anti-TrkA (1:200; Santa Cruz), anti-GAP43 (1:300; Santa Cruz), anti-NG2 (1:400; Proteintech), anti-Brevican (1:300; Proteintech), anti-5-HT (1:200; Abcam), anti-TMEM119 (1:200; Santa Cruz) at 4 °C overnight. After washing three times with PBS, the sections were then incubated with secondary Alexa Fluor 488, 594, or 405 conjugated mouse, rabbit, or chicken antibodies (1:200, Invitrogen) at 4°C in darkness for 2 h. ultimately, the sections were stained with 4', 6-Diamidino-2-phenylindole (DAPI, Beyotime, 1:1000; C1006) and following rinsied thoroughly with PBS. Image acquisition of Samples was conducted with a fluorescent microscope (DMi8 Thunder, Leica). The analysis were used the software LASX 3.5.1 and ImageJ version 1.51.

### TEM analysis and SEM

To observe the myelin regeneration, three microliters of diluted samples from different groups were separately applied to electron microscopy grids that were coated with a thin layer of continuous carbon film. Subsequently, these grids underwent staining with 2% (w/v) phosphotungstic acid. The samples were then visualized using a Tecnai Spirit 120 microscope (Thermo Fisher Scientific, USA) functioning at 120 kV. For the spinal cord lesion sites, transverse cuts were made and fixed with 2.5% glutaraldehyde at 4 °C for 24 hour. Ultrathin slices (80 nm thickness) were obtained using an ultramicrotome (Reichert E Co, Vienna, Austria). The slices were stained, polymerized, and imaged using a transmission electron microscope (Philips CM10, Eindhoven, Holland). Each group consisted of six rats. A total of 80 nerve fibers from 10 images chosen at random from each group were examined to assess neural morphology 44. We utilized ImageJ 1.51 to measure the density of myelinated axons, the area of axons, and the myelin present within individual axons. To investigate the surface structure of GelMA-AFN, we conducted observations by a scanning electron microscope (S-3400N; HITACHI, Japan) with an acceleration voltage set to 6 kV.

### EMG recording and processing

In order to to assess hindlimb electromyography (EMG), Implantation of electrodes were took place 8 weeks following SCI. This involved a non-damaged chip electrode made up of two receiving element. In a suitable environment, the chip electrodes were affixed to the gastrocnemius and tibial muscles of the rats when they were sober. As the rats moving freely, the electrical signal analyzer captured electrical signals. Subsequently, the muscle activity of the hindlimb was recorded on an oscilloscope, while the osteoarticular structures were monitored to identify phases of stance and swing. The EMG recording and processing were carried out using MATLAB by technicians who were unaware of the animal groups' assignments. In the investigation, we utilized the Poincaré analysis to discern chaos from randomness. Use the following formula to calculate a time series of EMG peak signals:

xt, x(t+1), x(t+2),...,x(t+n)

To create a return map of its simplest form, we plotted (xt, x(t+1)), (x(t+1), x(t+2)), and then (x(t+2), x(t+3)), and so forth. Xt is the signal value of the electromyographic signal x at time t, where t = 1, 2,…, t + n are time points separated by n time intervals from time t, where n = 1, 2,…. This methodology facilitated a clear depiction of the duration between two EMG peak signals within a sequence, allowing for the quantification of rhythm across various groups. Eventually, we extracted amplitude and rhythm data of the EMG information. In the Poincaré plot, a flatter ellipse is indicative of healthy individuals, while those who are unhealthy demonstrate a greater length-to-width ratio 45.

### RNA sequencing analysis

TRIzol was used to extract RNA from the lesion core of spinal cord. Subsequently, the quality of the RNA was assessed by the 5300 Bioanalyser (Agilent) and quantified using the ND-2000 (NanoDrop Technologies). RNA purification, reverse transcription, library construction, and sequencing were performed at Shanghai Bo Hao Biotechnology (China). The RNA sequencing library was processed according to Stranded mRNA Prep kit, Ligation from Illumina (San Diego, USA), application amount was 1 μg RNA. Following quantification using Qubit 4.0, the paired-end RNA-seq sequencing library was processed on the sequencer (Illumina, USA) at a read length of 2 × 150bp. The initial paired-end reads were subjected to trimming and quality evaluation using fastp with the default configurations. Next, the clean reads were aligned individually to the reference genome in orientation mode using HISAT2 software. For each sample, the aligned reads were then assembled through a reference-based method with StringTie.

### Quantitative qPCR analysis

The RNA extraction was performed using identical methods and sourced from the same origins as that utilized for RNA sequencing analysis. Using a spectrophotometer (Aglient, G9821A, USA) to determine the concentration and purity of the RNA. Subsequently, cDNA synthesis was performed from 1 μg RNA using the PrimeScript RT kit (TaKaRa). Specific primers were designed based on the target gene sequences, and PCR amplification was conducted with SYBR Premix Ex Taq II (TaKaRa) on a real-time PCR instrument (Applied Biosystems) under the following conditions: The complete cycle consists of 40 repetitions, which include initial pre-denaturation at 95 °C for 30 s, denaturation at 95 °C for 5 s, and annealing/extension at 60 °C for 34 s. The relative expression levels of the target genes were calculated using the 2^-ΔΔCT^ method, with conducted in triplicate.

### Statistical analysis

Statistical analyses were conducted using SPSS version 18.0 and GraphPad Prism version 9.0. The results are expressed as mean values with standard deviation (SD). A one-way analysis of variance (ANOVA) was employed for comparing multiple groups, whereas the t-test was utilized for comparing pairs of groups. Statistical significance was defined as *p < 0.05, **p< 0.01.

## Results

### Design and fabrication of a droplet microfluidic chip for producing GelMA-AFN microspheres

**Figure 1:**
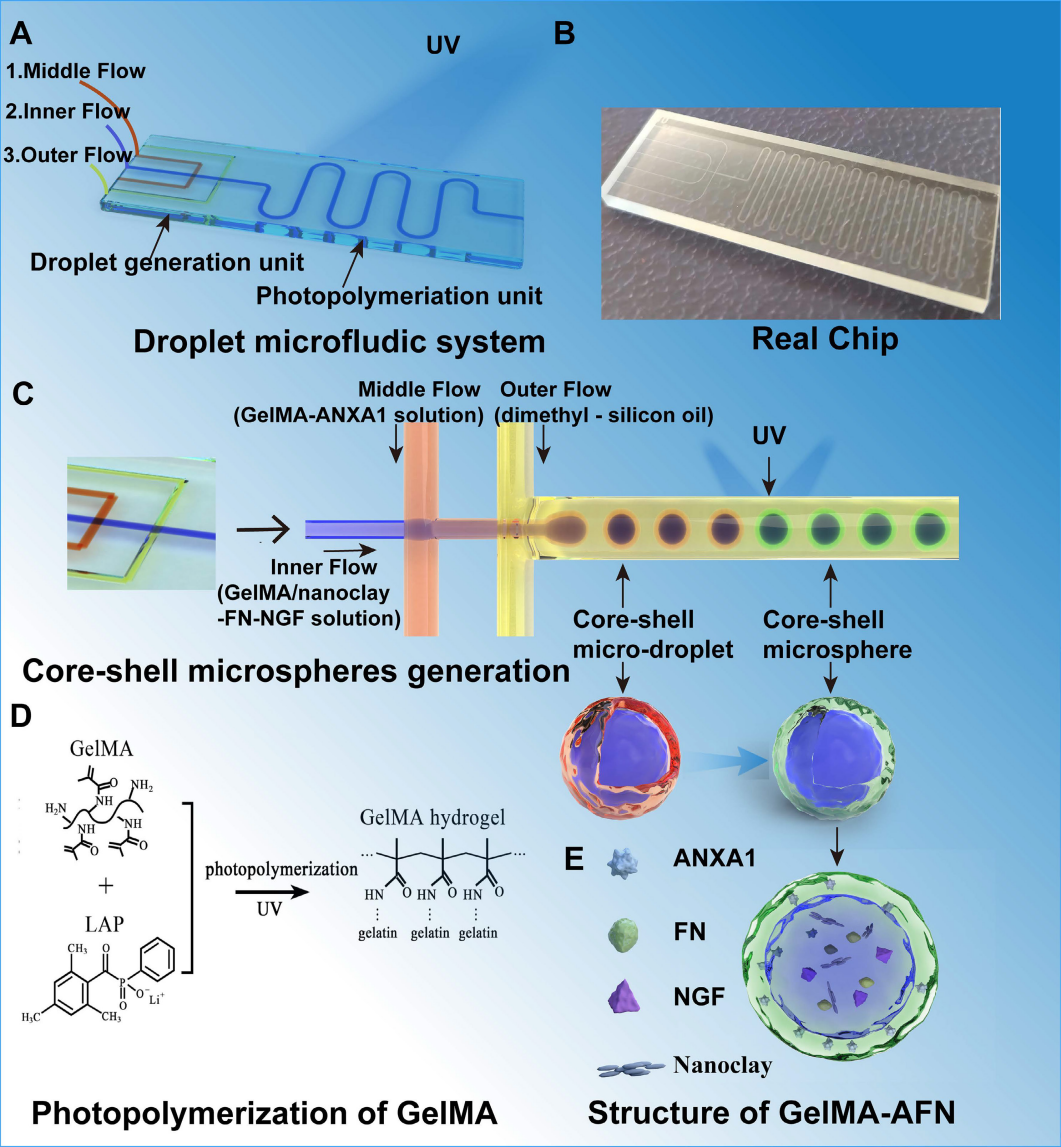
** This schematic diagram provides an overview of our microfluidic chip and the process for preparing double-layer microsphere GelMA-AFN. (A)** The droplet microfluidic system. **(B)** The real chip. **(C)** The generation of Core-shell microspheres. The inner, middle, and outer flows formed a double-layer droplet under the action of liquid shear force, then solidified into a double-layer microsphere with a core-shell structure after ultraviolet irradiation. **(D)** The photopolymerization of GelMA hydrogel. **(E)** The structure of GelMA-AFN.

We developed a droplet microfluidic chip to produce bilayer GelMA-AFN microgels. The chip comprised a microgel photopolymerization unit and a unit for generating droplets. (Figure 1A). The chip was designed as a flow-focusing microfluidic structure, the droplet generation unit ensures controlled fluid flow through various channels (Figure 1B). By utilizing a water-in-water-in-oil (w/w/o) system, microdroplets were generated through the shear force applied by the continuous flow. Within this w/w/o emulsion chip, the inner phase (GelMA/Nanoclay-FN-NGF solution), middle phase (GelMA-ANXA1 solution), and outer phase (dimethyl-silicon oil) were introduced via inlets 1, 2, and 3, respectively. The microgel photopolymerization unit features a serpentine channel designed to facilitate the formation of bilayer microgels upon UV irradiation. The degree of GelMA photopolymerization on microfluidic chips depends on the UV exposure time and intensity (Figure 1C). According to our pre-experimental results, an exposure time of 20s was selected as optimal. The microfluidic design enabled the controlled generation of monodispersed bilayer GelMA microgels. The principle of photopolymerization of GelMA with LAP (lithium phenyl-2,4,6-trimethylbenzoylphosphinate) and the concrete structure of GelMA-AFN was shown in the figure (Figure 1D, E).

### Characterization of GelMA-AFN

After conducting multiple experiments, the optimal flow rates for the preparation of GelMA-AFN were established. Individual microspheres were observable via optical microscopy at an inner phase velocity of 100 μL/min, a middle phase velocity of 150 μL/min, and an outer phase velocity of 200 μL/min. The production of monodispersed bilayer GelMA was controllable due to the microfluidic design. The microspheres, consistently sized with diameters of 50 μm, displayed uniform morphology upon observation.

**Figure 2:**
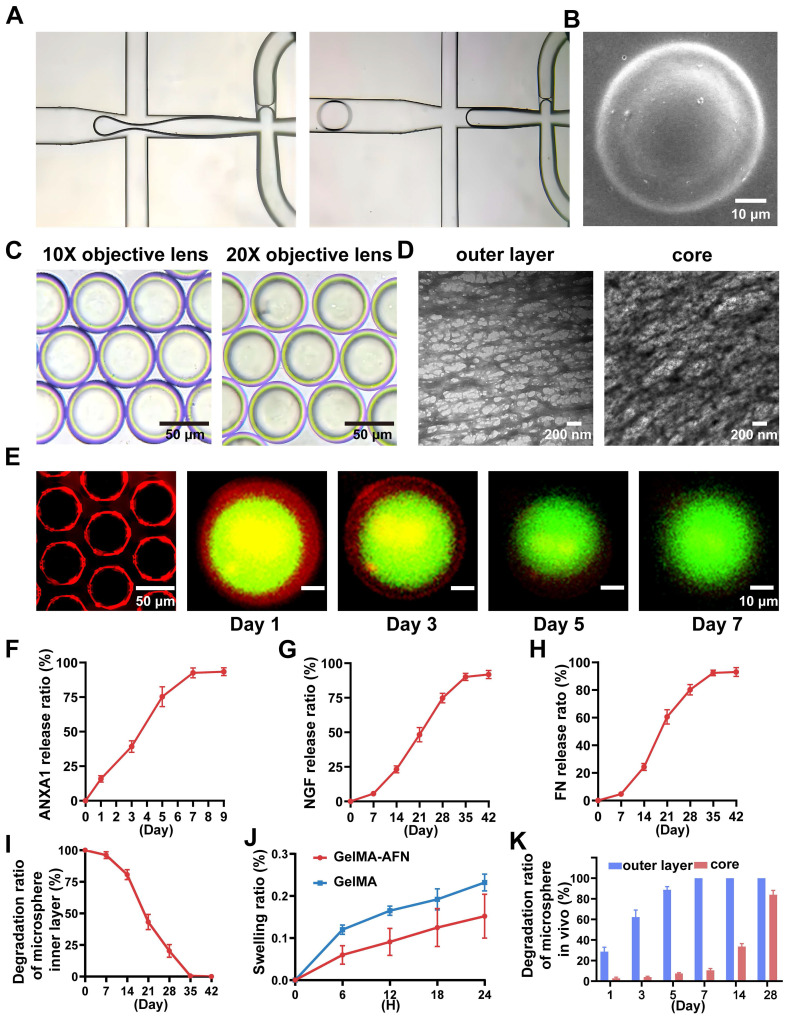
** Fabrication and characteristics of GelMA-AFN. (A) Preparation of GelMA-AFN microspheres in the microfluidic chip under light microscope (LM)**. **(B)** GelMA-AFN microspheres observed by scanning electron microscope (SEM). **(C)** GelMA-AFN microspheres observed by LM (10× ocular lens). **(D)** The GelMA hydrogels with the same composition as GelMA-AFN were observed by transmission electron microscope (TEM). **(E)** GelMA-AFN with rhodamine in its outer layer (red) and FITC in its inner layer (green) observed by fluorescence microscope (FM). **(F-H)** The release ratio of ANXA1 for 9 days and the release ratio of NGF, FN for 42 days. **(I)** The degradation ratio of inner layer of GelMA-AFN in PBS for 42 days. **(J)** The swelling ratio of GelMA-AFN and GelMA microsphere with the same concentration as GelMA-AFN for 24 h in PBS (50 μm, *n* = 6).** (K)** The degradation ratio of inner layer and core of GelMA-AFN *in vivo* for 28 days (*n = 6*).

Under the influence of shear force, the solution forms bilayer droplets as it passes through the two intersections of the microfluidic chip (Figure 2A). Following UV irradiation, these droplets appear as uniform bilayer microspheres with a diameter of 50 μm under a scanning electron microscope and light microscope (Figure 2B, C). Transmission electron microscopy of the hydrogels, which had the same composition as the inner and outer layers of GelMA-AFN, revealed that the core exhibited a denser structure due to the incorporation of nanoclay compared to the outer layer (Figure 2D). Large quantities of GelMA-AFN microspheres were prepared by adding rhodamine fluorescent stain to the outer microspheres. The inner microspheres were prepared without any fluorescent dye. Fluorescence microscopy revealed that the microspheres exhibited a distinct layered structure, characterized by intense red fluorescence in the outer layer and intense green fluorescence in the inner layer (Figure 2E). This confirms that the structure of microspheres aligns with the experimental design.

Adequate samples of GelMA-AFN were immersed in PBS and incubated for a duration of 24 h. Following the removal of surface moisture, the swollen microspheres were quantitatively measured at designated time. The expansion ratios of GelMA-AFN at 6, 12, 18, and 24 h were 5.99 ± 2.21%, 9.32 ± 3.18%, 12.50 ± 4.97%, and 15.84 ± 5.62%, respectively. In comparison, the expansion of GelMA-AFN without nanoclay was 12.74 ± 2.05%, 17.57 ± 3.33%, 20.63 ± 3.92%, and 24.63 ± 3.77% at the corresponding time points. The results showed that GelMA-AFN had consistently lower expansion ratios at all time points compared to formulations without nanoclay (Figure 2J). This suggests that nanoclay enhances cross-linking, improving structural integrity and resistance to deformation* in vivo*.

In addition, the drug encapsulation efficiency and drug loading capacity for the GelMA-AFN microspheres were determined. For the outer layer of the microsphere, the encapsulation efficiency of ANXA1 was 80.37 ± 5.08%, and the drug loading capacity was 21.54 ± 2.82%. For the inner layer of the microsphere, the encapsulation efficiency of NGF was 78.19 ± 6.02%, and the drug loading capacity was 8.54 ± 1.15%. Besides, the encapsulation efficiency of FN was 81.07 ± 4.83%, and the drug loading capacity was 7.83 ± 1.26%. Because the nanoclay occupied part of the loose pore structure of the inner Gelma gel of the microspheres, the combined drug loading of FN and NGF was less than that of ANXA1.

### Degradation properties and drug release of GelMA-AFN

GelMA-AFN microspheres, with shells containing rhodamine fluorescent stain, were immersed in PBS at 37°C. Shell degradation was monitored using fluorescence microscopy on days 1, 3, 5, 7, and 9. The results revealed that the degradation ratio of the shell was 16.23 ± 2.02% on day 1, 48.35 ± 5.67% on day 3, 72.19 ± 6.82% on day 5, 93.77 ± 4.31% on day 7, and 100% on day 9 (Figure 2E). These degradation ratios correspond to the timeline of inflammation development in early-stage SCI, indicating that the GelMA-AFN shell is an effective carrier for anti-inflammatory drugs.

The cumulative drug release ratio from GelMA-AFN was quantified by immersing the pellets in 1 mL PBS at 37 °C and analyzing them with an ELISA kit. The release ratios of ANXA1 on days 1, 3, 5, 7, and 9 were 15.83 ± 2.35%, 39.22 ± 4.15%, 75.33 ± 7.22%, 92.58 ± 3.62%, and 93.35% ± 2.83% respectively (Figure 2F). The release ratios of NGF on days 7, 14, 21, 28, 35, and 42 were 5.62 ± 1.09%, 23.19 ± 2.53%, 48.29 ± 5.31%, 74.83 ± 3.49%, 90.16 ± 2.58%, and 91.85 ± 2.98%, respectively (Figure 2G). The release ratios of FN on days 7, 14, 21, 28, 35, and 42 were 4.67 ± 1.25%, 24.33 ± 2.87%, 60.55 ± 5.17%, 80.17 ± 3.82%, 92.35 ± 2.02%, and 93.06 ± 3.15%, respectively (Figure 2H). The data indicated that the concentration of ANXA1 peaked on day 7, coinciding with the degradation of the microsphere. The release curves of NGF and FN were similar, with the concentration of NGF and FN peaking after complete degradation of the core of the microspheres, in line with the degradation characteristics described above.

Following the complete degradation of the GelMA-AFN shell, the remaining microspheres were immersed in PBS at 37 °C. The surface supernatant of the pellet was removed at specific time intervals. The results showed a degradation ratio of 3.69 ± 1.89% on day 7, 19.31 ± 3.90% on day 14, 51.52 ± 5.08% on day 21, 79.75 ± 4.29% on day 28, 99.48 ± 0.41% on day 35, and 100% on day 42 (Figure 2I). These findings suggest that the complete degradation time of the core of GelMA-AFN is more than one month, aligning with the critical period for effective nerve repair during SCI recovery. Thus, the core of GelMA-AFN serves as a suitable carrier for nerve-promoting repair drugs.

To further investigate the degradation of GelMA-AFN *in vivo*, we incorporated different fluorescent dyes into the outer and inner layers of the microsphere. Subsequently, the microsphere was injected into the spinal cord injury site of SCI rats, and the fluorescence intensity of the central tissue was analyzed on days 1, 3, 5, 7, 14, and 28. The degradation percentages of GelMA-AFN were recorded as follows: at days 1, 3, 5, 7, 14, and 28, the degradation ratios of the outer layer were 28.63 ± 4.28%, 62.29 ± 6.78%, 88.72 ± 3.16%, 100%, 100%, and 100% respectively. The degradation ratios of core were 2.75 ± 1.10%, 3.93 ± 0.87%, 7.45 ± 0.77%, 10.56 ± 1.65%, 33.61 ± 2.80%, and 83.94 ± 4.20%, respectively (Figure 2K, Figure S1B, C). In comparison, the degradation of GelMA-AFN in PBS solution was slightly faster.

### Neuroprotective effects and axonal growth induced by GelMA-AFN *in vitro*

**Figure 3:**
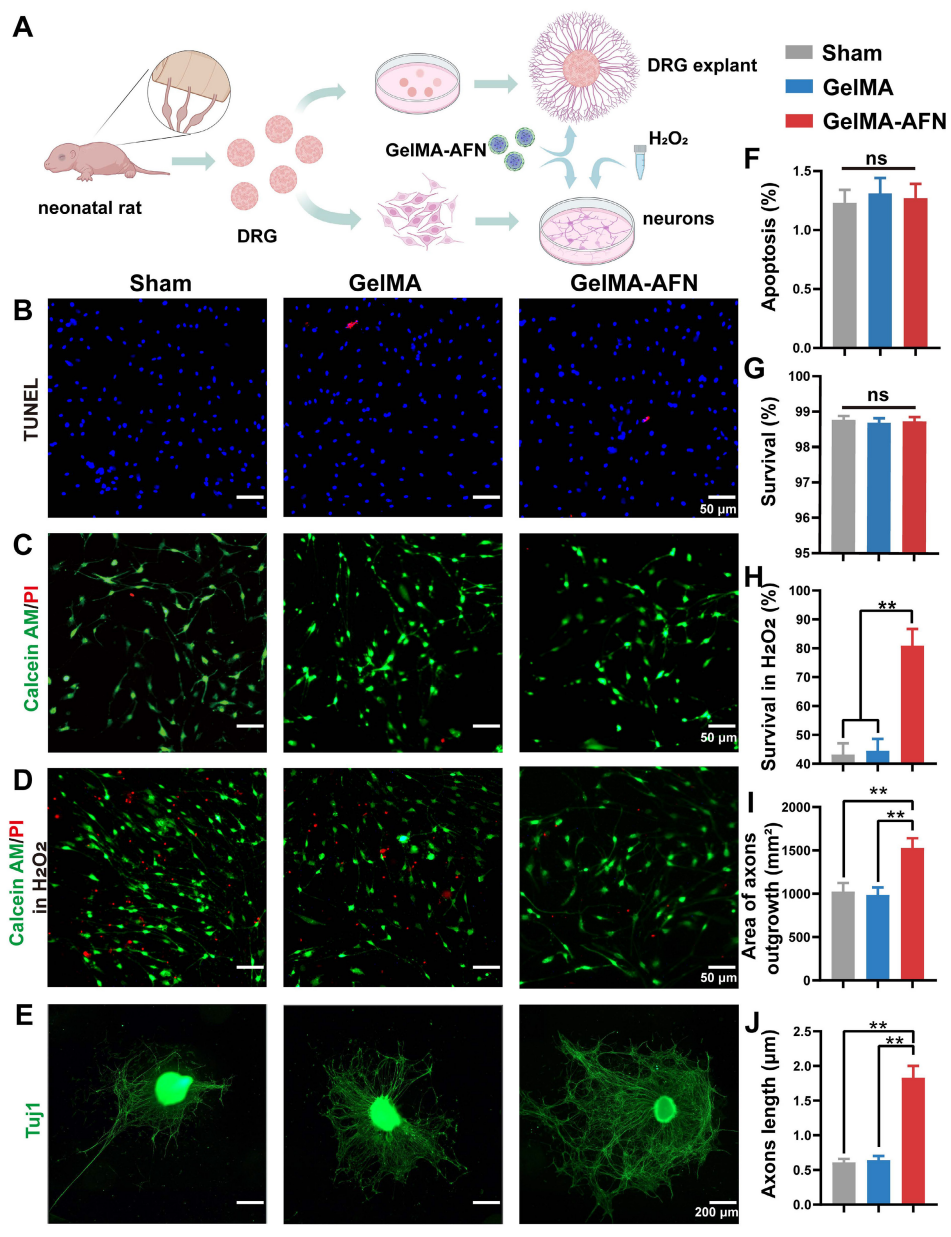
** Cytotoxicity of GelMA-AFN, GelMA-AFN reduced the death of neurons under the condition of H_2_O_2_ *in vitro* and promoted axon outgrowth of DRG explants. (A)** Extraction and culture of DRG neurons and DRG explants. **(B)** Representative fluorescence images of TUNEL (in red; marker of apoptotic cells) and DAPI (in blue; nuclear marker for both survival and apoptotic cells) stained neurons (scale bar: 50 µm). **(C)** Representative fluorescence images Calcein AM/PI staining of live neurons (green) and dead neurons (red) (scale bar: 50 µm). **(D)** Representative fluorescence images of Calcein AM/PI staining of live neurons (green) and dead neurons (red) under the condition of H_2_O_2_ (scale bar: 50 µm). **(E)** Tuj1 immunofluorescence staining images of DRG explants (scale bars: 200 µm). F-J) Comparisons of the cellular activity. **(F)** Apoptosis ratio (for Figure 3B), **(G)** Survival ratio (for Figure 3C), **(H)** Survival ratio in H_2_O_2_ (for Figure 3D), **(I)** DRG explant axons area (for Figure 3E), **(J)** DRG axon length (for Figure 3E). (**p < 0.01, *n* = 6).

To assess the potential safety of GelMA-AFN, we peformed an *in vitro* test to assess its effect on DRG neurons. Survival and apoptosis ratios were measured in three groups using live/dead assays and TUNEL staining (Figure 3A). The apoptosis ratios were found to be 1.23 ± 0.09% in the Sham group, 1.31 ± 0.11% in the GelMA group, and 1.27 ± 0.12% in the GelMA-AFN group, with no significant difference between the groups (Figure 3B, F) (p> 0.05). The survival ratios were found to be 98.77 ± 0.16% in Sham group, 98.69 ± 0.13% in GelMA group, and 98.73 ± 0.14% in GelMA-AFN group, with no significant difference between the groups (Figure 3C, G) (p> 0.05).

Additionally, the protective effect of GelMA-AFN on DRG neurons in an inflammatory environment was evaluated using a hydrogen peroxide model. The findings indicated a notable rise in the number of dead cells in all three groups (Figure 3D), with survival ratios below 50% in both the Sham and GelMA groups. However, the proportion of surviving neurons in the GelMA-AFN group (82.96 ± 3.75%) was significantly greater compared to the Sham and GelMA groups. This survival ratio was 1.87 times higher than the Sham group and 1.82 times higher than the GelMA group, respectively (Figure 3H) (p < 0.01). This suggests that GelMA-AFN releases ANXA1 promptly, reduces neuronal loss in an inflammatory environment, protects neuronal survival, and facilitates nerve repair.

We further investigated the effect of GelMA-AFN on axonal growth using DRG explants. After several days of co-culture, DRG explant axons were labeled with Tuj1 (Figure 3E). The results revealed that the average axon length for the Sham and GelMA groups were 1024.37 ± 101.25 μm and 987.53 ± 89.77 μm. Furthermore, the area taken up by axons in DRG explants was 0.61 ± 0.05 mm^2^ in the Sham group, comparing 0.64 ± 0.06 mm^2^ in the GelMA group, showing no significant difference between the two groups (p > 0.05). However, the GelMA-AFN group showed a obvious advantage in all these measurements. The average axon length for DRG explants in this group was 1529.13 ± 112.63 μm, which was 1.50 times and 1.55 times greater than that of the Sham and GelMA groups (Figure 3J) (p < 0.01). Moreover, the area occupied by axons was 1.63 ± 0.17 mm^2^, which was 2.67 times than the Sham group and 2.55 times than the GelMA group (Figure 3I) (p < 0.01). The results indicate that GelMA-AFN releases FN and NGF, which act as essential adhesive substrates and provide nutrients for axon growth, thereby promoting axon growth.

### The effect of GelMA-AFN on neutrophils and macrophages in modulating Inflammation after SCI

Acute SCI often triggers inflammation, which is a key driver of extensive neuronal death. Mitigating this damage may involve reducing the number of macrophages in the injured region. This could minimize the necrotic cavity in the tissue and slow glial cell proliferation near the injury part. The inflammatory response in the SCI area leads to the recruitment of macrophages *in vivo* 46. To investigate the potential of GelMA-AFN in regulating immune cell subtypes and their recruitment following acute SCI, spinal cord tissue was extracted from rats 7 days after surgery and subjected to flow cytometric analysis 47.

**Figure 4:**
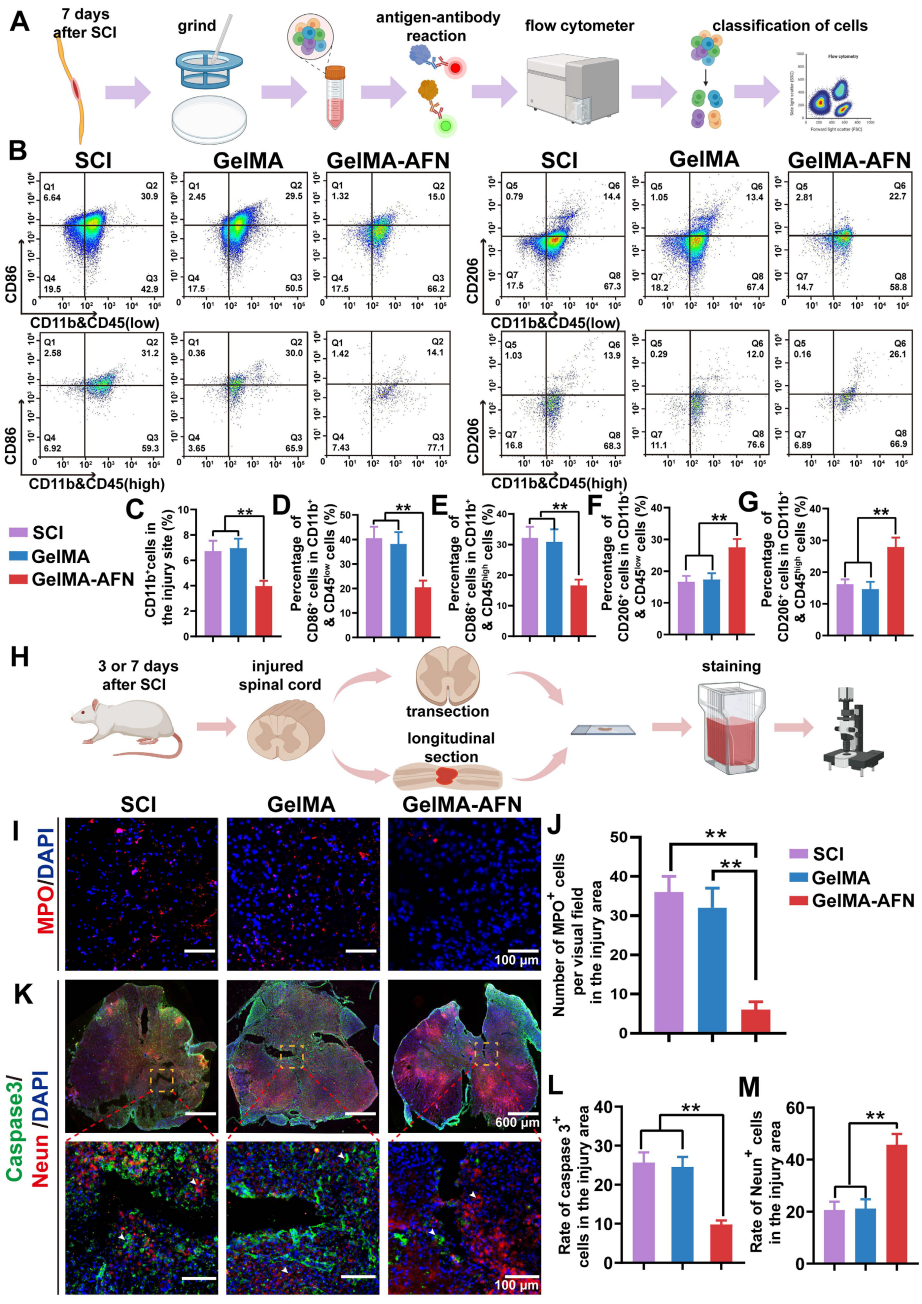
** GelMA-AFN promoted cell survival by reducing early inflammation and inhibiting apoptosis *in vivo*. (A)** Flow cytometry process. **(B)** Flow ratecytometric analysis of CD11b, CD45, CD86 and CD206 cells at the injury site, categorized into macrophages (CD11b^+^ & CD45^high^) and microglia (CD11b^+^ & CD45^low^). *n* = 6. **(C-E)** The number of positive cells at the injury site. **(C)** CD11b^+^, **(D)** CD11b^+^ & CD45^low^ cell positive for CD86 (M1 microglia), **(E)** CD11b^+^ & CD45^high^ cells positive for CD86 (M1 macrophages),** (F)** CD11b^+^ & CD45^low^ cells positive for CD206 (M2 microglia), **(G)** CD11b^+^ &CD45^high^ cells positive for CD206 (M2 macrophages). **(H)** Procedure of spinal cord section and immunofluorescence staining. **(I)** Representative immunofluorescence images from the lesion center of longitudinal spinal cord sections, stained with anti-MPO (red, neutrophils) and DAPI (blue, nuclear marker) (scale bar: 100 µm). **(J)** Quantification of MPO^+^ cells per visual field in the injury area. **(K)** Representative immunofluorescence images of spinal cord sections stained with anti-Caspase-3 (green, apoptotic cells), anti-NeuN (red, neuronal nuclei), and DAPI (blue, nuclear marker) (scale bar: 600 µm, 100µm). **(L, M)** Quantification of Caspase-3^+^, Neun^+^ cells in the injury area (**p < 0.01, *n* = 6).

Both macrophages and microglia are CD11b-positive and distinguished by CD45 expression, with macrophages being CD45^high^ and microglia CD45^low^ (Figure 4B) 48. Subsequently, the cell populations were additionally categorized into M1 subtypes (CD86^+^), which are pro-inflammatory, and M2 subtypes (CD206^+^), known for their anti-inflammatory properties (Figure 4B). Flow cytometry results revealed significant recruitment of macrophages and microglia in both the SCI and GelMA groups, with CD11b^+^ cells at 6.73 ± 0.61% and 6.95 ± 0.75%, respectively, while the GelMA-AFN group had a lower proportion of 3.97 ± 0.41% (Figure 4C). The proportions of CD11b^+^ CD45^low^ and CD86^+^ cells were similar between the SCI (40.53 ± 4.67%) and GelMA groups (38.17 ± 4.83%) with no significant difference (p > 0.05). In the GelMA-AFN group, this proportion was significantly reduced to 20.47 ± 2.72%, representing 50.51% of the SCI group and 53.63% of the GelMA group (Figure 4D). The proportions of CD86^+^ in CD11b^+^ CD45^high^ cells were 32.15 ± 3.67% in the SCI group and 30.87 ± 4.09% in the GelMA group (p > 0.05). In the GelMA-AFN group, this proportion was 16.63 ± 1.91%, which accounted for 51.73% of the results in the SCI group and 53.87% in the GelMA group (p < 0.01, Figure 4E). In contrast, the proportions of CD206^+^ in CD11b^+^ CD45^low^ cells in the GelMA-AFN group (27.51 ± 2.62%) were significantly higher than in the SCI (16.63 ± 1.88%) and GelMA (17.33 ± 2.02%) groups (p < 0.01, Figure 4F). A semblable trend was observed for the ratio of CD206^+^ in CD11b^+^&CD45^high^ cells, with the GelMA-AFN group (27.89 ± 2.97%) showing a significant increase compared to SCI (16.18 ± 1.53%) and GelMA (14.64 ± 2.25%) groups (p < 0.01, Figure 4G). These findings suggest that GelMA-AFN promoted a shift from M1 to M2 macrophages and microglia, reducing inflammation and enhancing neuronal survival in the injured area. The controlled release of ANXA1 by GelMA-AFN played a key role in decreasing the macrophage count and modulating their phenotype, favoring an anti-inflammatory M2 response.

Within the first 1 to 3 days after SCI, neutrophils significantly infiltrate the injured area. This infiltration disrupts the blood-spinal cord barrier, leading to the release of various pro-inflammatory mediators, exacerbating spinal cord tissue damage 49. This secondary damage may lead to considerable neuronal loss, exacerbate tissue damage, and subsequently impact the reconstruction of synapses and recovery of neural circuits. Since the central cavity had not yet formed in the injured area during this time, neutrophil infiltration in each group of rats was evaluated by comparing spinal cord slices taken 3 days post surgery. The Sham and GelMA groups showed significant neutrophil infiltration, with 36.35 ± 3.72 and 32.17 ± 4.58 neutrophils per field (p > 0.05). In contrast, neutrophil infiltration in the GelMA-AFN group was minimal, with only 6.21 ± 1.59 neutrophils per field of view (Figure 4I, J). This suggests that GelMA-AFN released ANXA 1 during the early stages of SCI, effectively inhibiting neutrophil infiltration.

### GelMA-AFN activates the FPR2 receptor, modulates cytokines, and reduces apoptosis of cells after SCI

**Figure 5:**
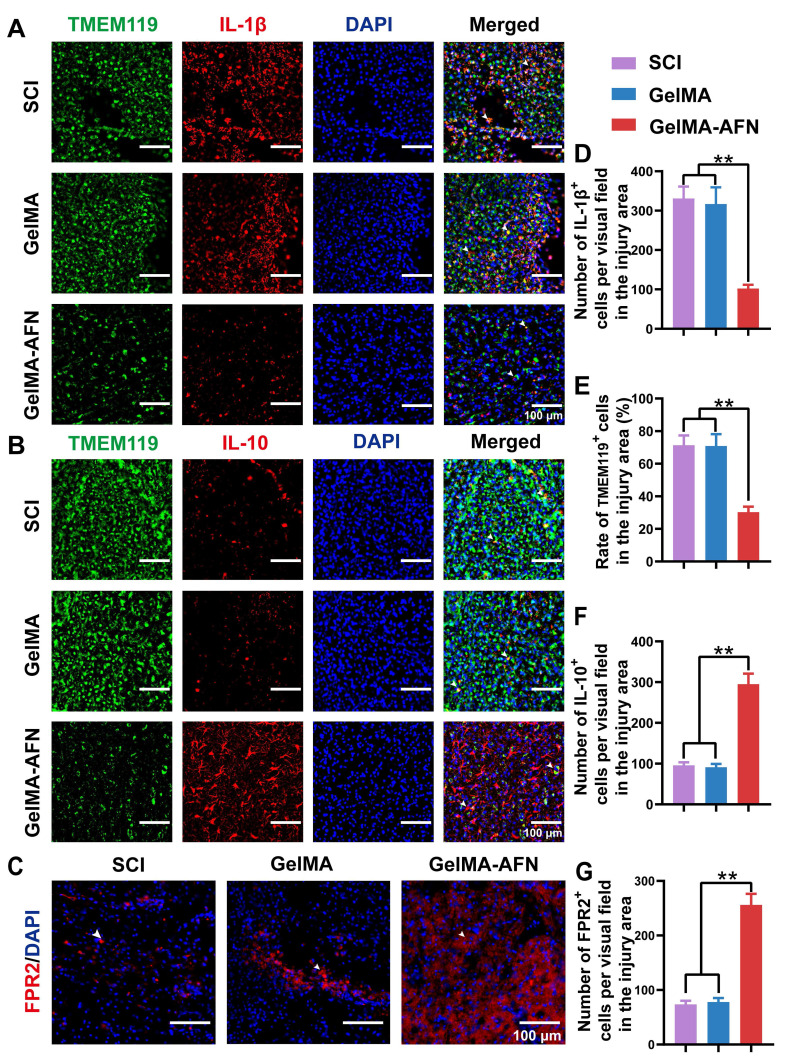
** GelMA-AFN activated FPR2 receptor, modulated cytokines. (A-C)** Immunofluorescence representative magnified images from the injury site of longitudinal spinal cord sections 7 days post SCI. The spinal cords were stained with **(A)** anti-TMEM119 (in green, marker of microglia), anti-IL-1β (in red), and DAPI (in blue, nuclear marker) (scale bar: 100 µm). **(B)** anti-TMEM119 (in green, marker of microglia), anti-IL-10 (in red), and DAPI (in blue, marker of nuclear marker) (scale bar: 100 µm). **(C)** anti-FPR2 (in red, marker of ANXA1 receptor) and DAPI (in blue, nuclear marker) (scale bar: 100 µm). **(D)** Mean IL-1β^+^ cells per visual field in the injury area.** (E)** The ratio of TMEM119^+^ cells in the injury area. **(F)** Mean IL-10^+^ cells per visual field in the injury area. **(G)** Mean FPR2^+^ cells per visual field in the injury area (**p < 0.01, *n* = 6).

Given the neuroprotective effects of ANXA1 in attenuating the inhibitory microenvironment *in vitro*, we hypothesized that ANXA1 could mitigate inflammation and reduce apoptosis in the context of SCI. Seven days post-injury, Caspase-3 immunostaining revealed no obvious difference in the ratio of Caspase-3-positive cells between the SCI group (25.67 ± 2.61 %) and GelMA group (24.58 ± 2.53 %) (p > 0.05). Nevertheless, both the SCI and GelMA groups exhibited conspicuous higher apoptosis ratios compared to the GelMA-AFN group (9.83 ± 1.02 %, p < 0.01, Figure 4H, K, L). Specifically, the SCI and GelMA groups had approximately 2.61-fold and 2.50-fold higher apoptosis ratios, respectively, compared to the GelMA-AFN group. Furthermore, the GelMA-AFN group showed a significantly higher proportion of Neun-positive cells (45.73 ± 4.15 %) within the lesion area, more than twice the counts observed in both the GelMA (21.18 ± 3.57 %) and SCI (20.64 ± 3.19 %) groups (p < 0.01, Figure 4K, M). These findings strongly suggest that ANXA1 effectively inhibits apoptosis and preserves neuronal integrity during the acute phase of SCI. To further investigate the anti-inflammatory effects of ANXA1, we analyzed microglial activation and inflammatory cytokine levels during acute stage of SCI. Seven days post-injury, TMEM119 immunostaining showed no significant difference in microglial activation between the SCI (71.27 ± 6.13 %) and GelMA groups (70.88 ± 7.26 %) (p > 0.05). On the contrary, the GelMA-AFN group displayed evidently lower than microglial activation (30.31 ± 3.27 %), indicating a substantial reduction in neuroinflammation (p < 0.01, Figure 5A, E). This trend was consistent with the cytokine expression profiles. The pro-inflammatory cytokine IL-1β exhibited the lowest level in the GelMA-AFN group (102.17 ± 9.73), corresponding to only 0.31 times the level observed in the SCI group (331.57 ± 39.83) and 0.32 times that in the GelMA group (317.15 ± 40.91) (p < 0.01, Figure 5A, D). Conversely, the anti-inflammatory cytokine IL-10 was most highly expressed in the GelMA-AFN group (295.33 ± 25.46) positive cells per visual field, approximately 3.10 times higher than the SCI group (95.67 ± 7.53) and 3.24 folds than the GelMA group (91.16 ± 8.12) (p < 0.01, Figure 5B, F).

Additionally, the number of FPR2-positive cells per field in the GelMA-AFN group (256.17 ± 20.15) was significantly higher than in both the SCI (73.50 ± 6.87) and GelMA (78.00 ± 7.33) groups, showing 3.48-fold and 3.28-fold increases, respectively (p < 0.01, Figure 5C, G). The findings indicate that the introduction of exogenous ANXA1 through GelMA-AFN microspheres effectively activated the FPR2 receptor at the injury site. Consequently, this activation suppressed microglial aggregation, reduced pro-inflammatory cytokines, increased anti-inflammatory cytokines and ultimately created a more favorable microenvironment for spinal cord recovery. Our experiments showed that in an inflammatory environment, ANXA1 effectively suppresses IL-1β secretion while promoting IL-10 release, thereby reducing apoptosis and protecting neuronal survival. These results suggest that ANXA1 mitigate the early inflammatory environment post SCI as well as reducing its detrimental effects on neurons.

### Gene expression changes induced by GelMA-AFN in SCI tissue revealed by RNA sequencing at 7 days post-SCI

**Figure 6:**
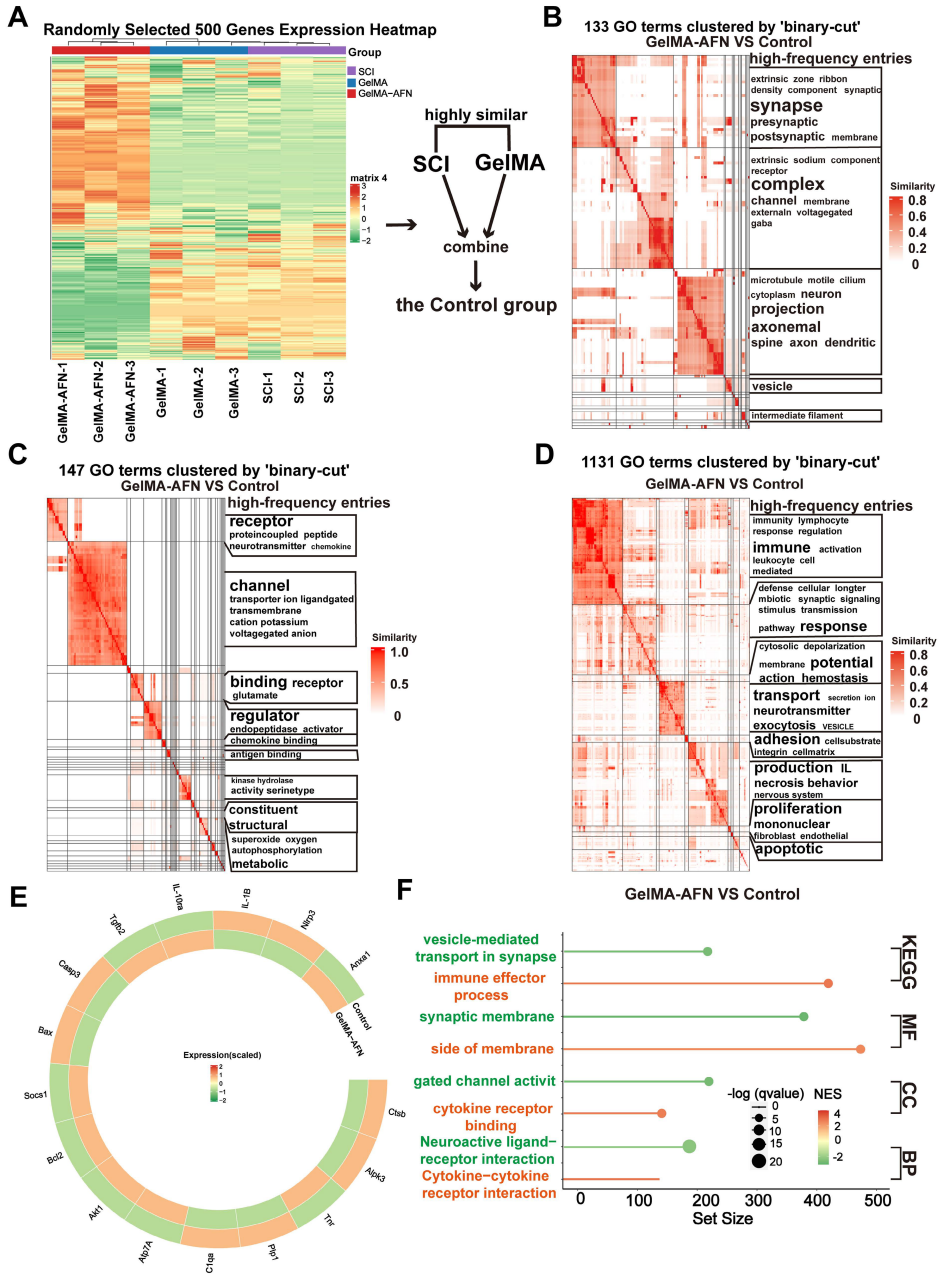
** Gene Expression Changes Induced by GelMA-AFN in spinal cord tissue 7 days post-SCI. (A)** Clustering heat map based on randomly selected 500 gene expressions. **(B)** Gene Ontology (GO) enrichment analysis of DEGs focused on Cell Component (CC). **(C)** GO enrichment analysis of DEGs focused on Biological Process (BP). **(D)** GO enrichment analysis of DEGs focused on Molecular Function (MF). **(E)** Circos heat map of 16 DEGs expression associated with inflammation. **(F)** The results of GSEA enrichment analysis between the GelMA-AFN and control groups show the top pathway with maximum and smallest NES (enrichment score) values in each category based on the GO (CC, MF, BP) and KEGG database. (|log2FC| > 1.5 and p < 0.05, *n* = 6).

In order to clarify the the alterations in gene within each group following SCI, total RNA was isolated from central spinal cord 7 days post-injury using TRIzol, followed by RNA sequencing. Differentially expressed genes (DEGs) were determined with a criterion of |log2FC| > 1.5 and p-value < 0.05. Clustering heatmap analysis showed no obvious difference in gene expression between the SCI group and the GelMA group, leading to their combination into a single Control group for comparison with the GelMA-AFN group (Figure 6A). Analysis of functional pathway enrichment through the Gene Ontology (GO) database revealed that DEGs in the biological processes category were mainly enriched in pathways related to inflammatory regulation, energy metabolism, cell adhesion, and membrane potential regulation. In the molecular function category, DEGs were enriched in ion channel activity, signal receptor activation, and receptor-ligand binding (Figure 6B, C, D). Gene Enrichment Set Analysis (GESA) showed significantly lower expression of inflammation-related pathways in the GelMA-AFN group compared to the Control group. In total, 1953 DEGs were identified, including 1061 downregulated and 892 upregulated genes. Notably, the expression levels of anti-inflammatory genes were markedly elevated in the GelMA-AFN group, demonstrating that GelMA-AFN effectively activates anti-inflammatory pathways in the tissue. Post-treatment with GelMA-AFN microspheres, cells secreted a good deal of anti-inflammatory cytokines, such as IL-10 and TGF-β, while the expression of pro-inflammatory cytokines was suppressed. Upregulation of the Bcl-2 gene inhibited the production and release of inflammatory mediators, suppressed apoptosis, and decreased neuronal loss. The upregulation of ATP7A enhanced cellular copper ion transport, leading to a decrease in the buildup of reactive oxygen species and free radicals. Additionally, the expression of genes associated with ANXA1 and its receptors was significantly increased, indicating that exogenous ANXA1 protein effectively executed its anti-inflammatory role at the early stage of the treatment. The upregulation of Akt1 and SOCS1 effectively inhibited the activation of inflammatory signaling, such as TLR4/Myd88/NF-κB. The expression of pro-inflammatory genes was significantly lower in the GelMA-AFN group than that of the Control group, confirming that GelMA-AFN mitigates inflammation in spinal cord. The synthesis and expression of pro-inflammatory cytokines, including IL-1β, and enzymes like CTSB, were inhibited, leading to the downregulation of NLRP3 inflammasome activation and suppression of its downstream cascade involving Caspase-3 and other enzymes. Downregulation of the Bax gene further inhibited apoptosis, increasing cell survival ratios. Concurrently, the inhibition of PLP1 gene expression contributed to reduced neuronal death (Figure 6E, F, Figure S3). Then we made use of PCR to detect the expression of ANXA1 and other genes in spinal cord tissue which 7 days post-SCI, and the results were consistent with the RNA Sequencing (Figure S2A).

### GelMA-AFN activates TrkA receptors and regulates the CSPG components, accelerating axonal outgrowth and suppressing glial scar formation

**Figure 7:**
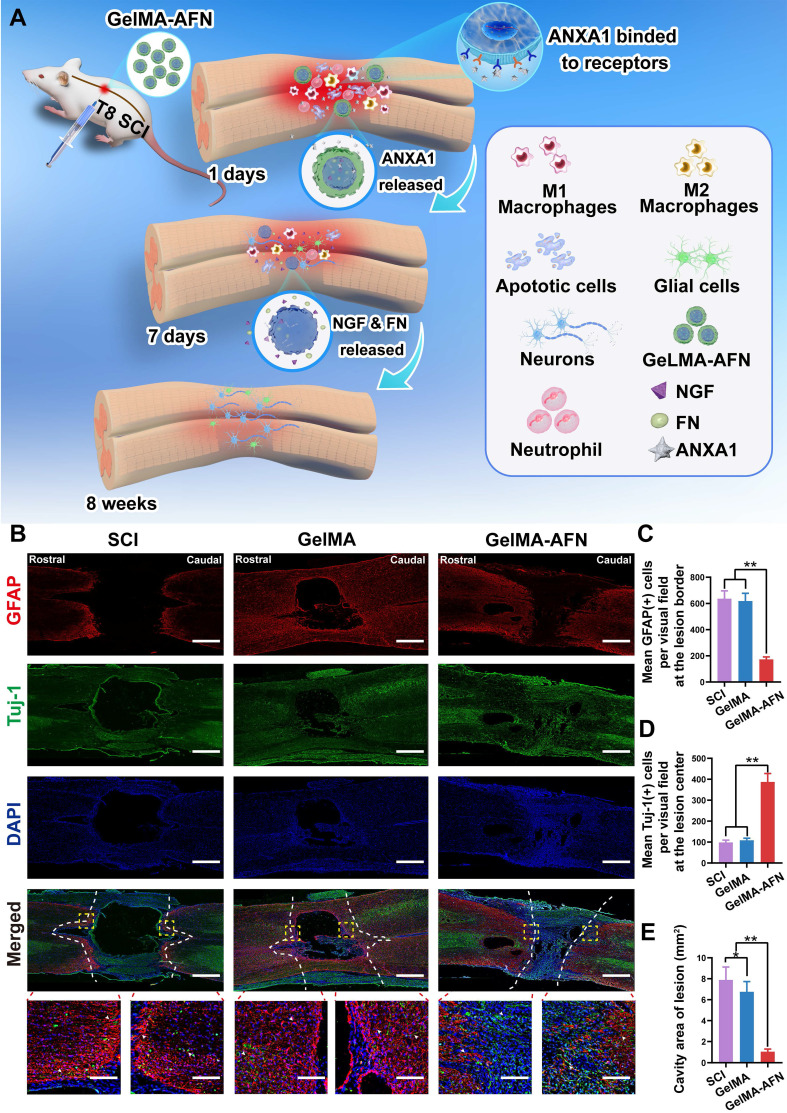
** The biological mechanism of GelMA-AFN and GelMA-AFN promoted nerve axons regeneration while reducing reactive astrogliosis formation *in vivo*. (A)** The biological mechanism of GelMA-AFN within 8 weeks after SCI. **(B)** Representative immunofluorescence images of rats at 8 weeks post-injury. The spinal cords were stained with antibodies against glial fibrillary acidic protein (GFAP, in red, marker of astrocytes), Tuj1 (in green, marker of neurons) and DAPI (in blue, marker of nuclear) (scale bar: 300 µm, 50 µm). **(C)** Mean GFAP^+^ cells per visual field in the injury area. **(D)** Mean Tuj1^+^ fluorescence area ratio per visual field in the injury area. **(E)** Cavity area of lesion (mm^2^) (**p < 0.01, *n* = 6).

Axonal growth and glial scar formation are crucial elements that impact recovery following SCI. This research focused on examining the capability of GelMA-AFN in accelerating nerve regeneration and inhibiting glial scar formation after SCI (Figure 7A). To assess this, we utilized Tuj1 labeling to identify neuronal axons and glial fibrillary acidic protein (GFAP) labeling to identify astrocytes. The number of GFAP^+^ cells and Tuj1^+^ fluorescence area ratio per visual field were used for assessing the expression levels of these markers within the SCI area. The expression of GFAP was signally reduced in the GelMA-AFN group (172.57 ± 18.63) when compared to both the SCI group (635.82 ± 60.23) and the GelMA group (618.36 ± 58.57) (p < 0.01). The mean GFAP^+^ cells per visual field in the GelMA-AFN group was 27.14% of the SCI group and 27.91% of the GelMA group (Figure 7B, C). The Tuj1 fluorescence positive area ratio per visual field showed no significant differences between the SCI group (4.33 ± 0.74%) and the GelMA group (3.95 ± 0.78%) (p > 0.05), indicating that drug-free GelMA microspheres did not promote neuronal axon growth.

In contrast, the GelMA-AFN group (20.92 ± 1.96%) exhibited the highest Tuj1^+^ fluorescence positive area ratio per visual field among the three groups, which was markedly higher than both the SCI group and the GelMA group, with values 4.83 times and 5.30 times greater, respectively (p < 0.01, Figure 7B, D). This suggests that axon regeneration of the damaged spinal cord was significantly enhanced following treatment with GelMA-AFN. The presence of GelMA-AFN greatly influences the growth of nerve axons and reduces the proliferation of astrocytes. This leads to a observably decrease in the formation of glial scar, which in turn promotes the distal growth of residual axons after SCI. The cavity area following tissue necrosis after SCI also showed significant differences evaluated by H&E staining (Figure S1D). The GelMA-AFN group (0.85 ± 0.02 mm^2^) had the lowest mean cavity area, compared to the SCI group (7.52 ± 0.81 mm^2^) and the GelMA group (6.86 ± 0.63 mm^2^). These values were 8.85 times and 8.10 times higher than those in the GelMA-AFN group (Figure 7E). This indicates that GelMA-AFN can effectively reduce the volume of the necrotic cavity after SCI (p < 0.01), thereby dumbing down the neural restoration. It is worth noting that the cavity area in the GelMA group was slightly smaller compared to the SCI group, the difference that may be due to the supportive and filling properties of the hydrogel microspheres (Figure S1D).

**Figure 8:**
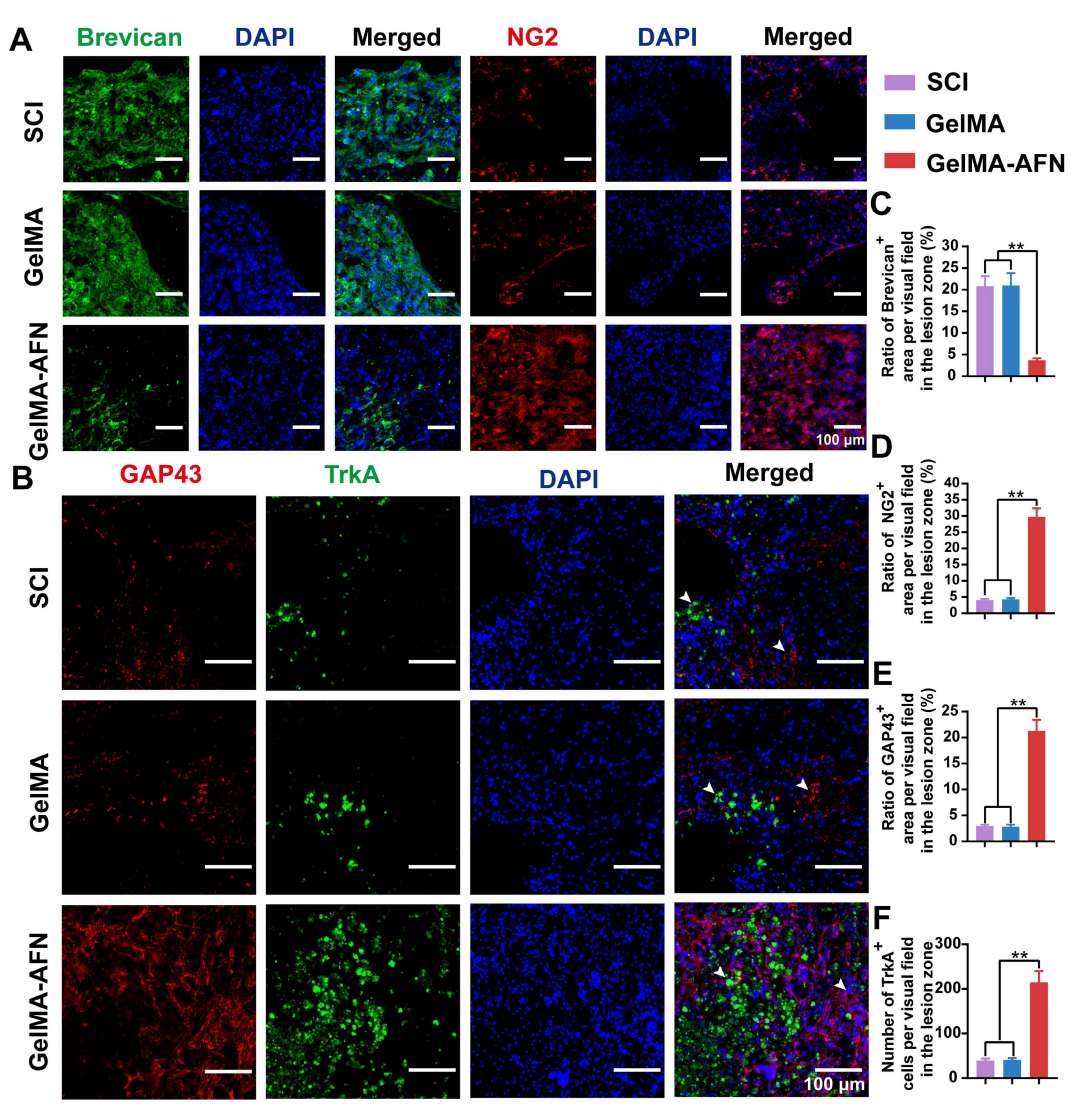
** GelMA-AFN regulated the CSPG components and activated TrkA receptors *in vivo*. (A)** Representative immunofluorescence images of injury site of longitudinal spinal cord sections at 8 weeks post-injury. The spinal cords were stained with anti-Brevican (in green, marker of chondroitin sulfate proteoglycans), anti-NG2 (in red, marker of chondroitin sulfate proteoglycans), and DAPI (in blue, marker of nuclear) (scale bar: 100 µm, 50 µm). **(B)** Representative immunofluorescence images of injury site of longitudinal spinal cord sections at 8 weeks post-injury. The spinal cords were stained with anti-GAP43 (in red, marker of regeneration axons), TrkA (in green, marker of NGF receptor), and DAPI (in blue, marker of nuclear) (scale bar: 100 µm). **(C-E)** The mean ratio of fluorescence positive area per visual field in the injury area. **(C)** brevican, **(D)** NG2, **(E)** GAP43.** (F)** Mean TrkA^+^ cells per visual field in the injury area. (**p < 0.01, *n* = 6).

Astrocytes produce chondroitin sulfate proteoglycans (CSPGs), which can either inhibit or support axonal growth, depending on the specific components involved. To investigate the effects of GelMA-AFN on CSPG secretion within the spinal cord, we conducted immunostaining to differentiate between inhibitory and supportive CSPG components. Notably, brevican, which inhibits axonal growth, and NG2, which promotes it, were the focus of our analysis at 8 weeks post-SCI (Figure 8A). The GelMA-AFN group showed a dramatically improve in NG2 expression and a marked decrease in brevican levels. Specifically, the number of brevican-positive area ratio per visual field in the GelMA-AFN group (3.69 ± 0.45%) was much lower compared to the SCI group (20.80 ± 2.38%) and the GelMA group (21.01 ± 2.83%) (p < 0.01, Figure 8A, C). In contrast, NG2 expression in the GelMA-AFN group (29.74 ± 2.66%) was 7.33 folds than in the SCI group (4.06 ± 0.36%) and 6.85 times higher than in the GelMA group (4.34 ± 0.38%) (p < 0.01, Figure 8A, D). These results indicate that GelMA-AFN effectively modulates CSPG composition, enhancing the expression of components that support axonal growth while suppressing those that inhibit it.

GelMA-AFN microspheres can continuously release exogenous NGF to the SCI site. To assess the activation of NGF receptors and the extent of axonal regeneration, we conducted TrkA and GAP43 staining 8 weeks post-SCI (Figure 8B). The results indicated that GelMA-AFN significantly activated NGF receptors in the SCI region. Plenty of newly formed axons were observed in the lesion core of the GelMA-AFN group, with an average GAP43^+^ area ratio per visual field (21.28 ± 2.12%), which was 7.24 times that of the GelMA group (2.94 ± 0.26%) and 7.52 times that of the SCI group (2.83 ± 0.40%) (p <0.01, Figure 8B, E). The average number of TrkA-positive cells per field in the GelMA-AFN group was (214.89 ± 25.32), which represents 5.23 times that of the GelMA group (41.12 ± 3.75) and 5.41 times that of the SCI group (39.70 ± 4.08) (p <0.01, Figure 8B, F). These results indicate that GelMA-AFN microspheres effectively deliver NGF to the SCI site, activate numerous NGF receptors, and significantly enhance the growth of new axons.

### GelMA-AFN increases neurological substrate content and promotes nerve fiber regeneration

**Figure 9:**
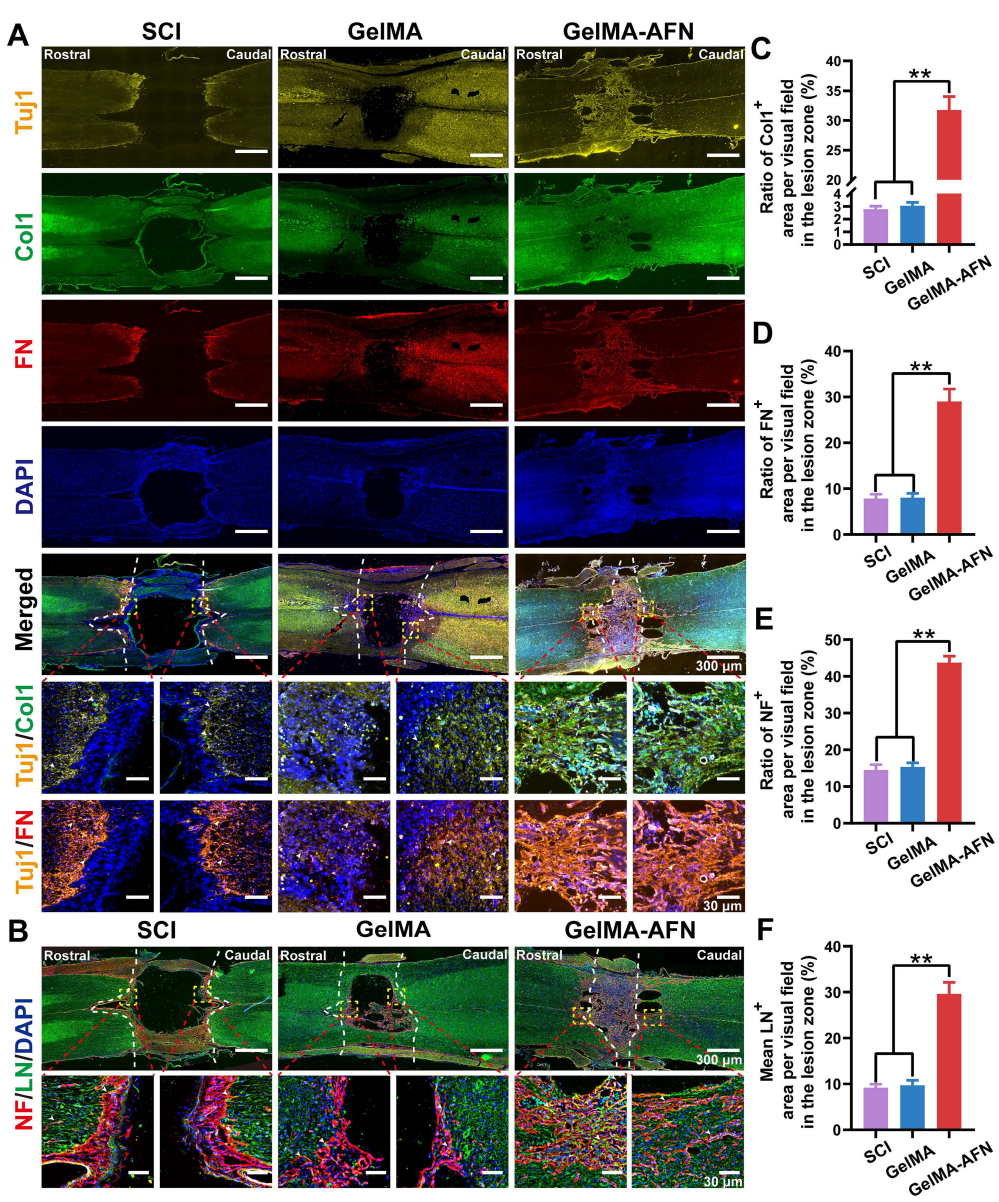
** GelMA-AFN increases neurological substrate content and promotes nerve fiber regeneration at 8 weeks post-injury. (A)** Representative immunofluorescence images of injury site. The spinal cords were stained with anti-Tuj1 (in yellow, marker of neurons), anti-Col1 (in green, marker of typeⅠ collagen), anti-FN (in red, marker of fibronectin), and DAPI (in blue, marker of nuclear) (scale bar: 300 µm, 30 µm). **(B)** Representative immunofluorescence images of injury site. The spinal cords were stained with anti-NF (in red, marker of neurofilament), anti-LN (in green, marker of laminin), and DAPI (in blue, marker of nuclear) (scale bar: 300 µm, 30 µm). **(C-F)** Mean ratio of fluorescence positive area per visual field in the injury area. **(C)** Col1, **(D)** FN, **(E)** NF, **(F)** LN (**p < 0.01, *n* = 6).

The ECM plays a pivotal role in nerve repair by providing a microenvironment that supports guided axonal growth. To evaluate whether neurons and associated ECM components develop concurrently after exogenous FN supplementation, we performed immunofluorescence staining on rat spinal cords 8 weeks post-injury. The findings displayed that, in the GelMA-AFN group, the injured spinal cord regions exhibited extensive co-localization of nerve fibers with ECM markers (Figure 9A). The Col1 positive area ratio was 11.42 times and 10.41 times greater in the GelMA-AFN group (31.76 ± 2.26%) compared to the GelMA (2.78 ± 0.22%) and SCI groups (3.05 ± 0.26%). Similarly, the FN positive area ratio per visual field was 3.61 times higher in the GelMA-AFN group (28.97 ± 2.74%) compared to the GelMA group (8.02 ± 0.96%), and 3.69 folds than that of the SCI group (7.85 ± 0.92%), respectively (p < 0.01, Figure 9A, C, D). These findings indicated that the sustained release of FN from GelMA-AFN microspheres significantly enhances ECM content within the injury site, thus establishing a microenvironment that supports axonal growth and promotes nerve regeneration.

Neuroregeneration is crucial for evaluating recovery from SCI. To determine whether GelMA-AFN benefits neurological substrate content and enhances nerve regeneration after SCI, this study utilized NF200 labeling to identify neurons and LN labeling to assess the expression level of laminin within the injured area (Figure 9B). Protein expression was quantified by calculating the mean positive expressing area ratio per visual field in the damaged area. The number of NF and LN positive area ratios per visual field in the SCI group was 14.53 ± 1.43% and 9.17 ± 0.79%, respectively.

In the GelMA group, the NF and LN positive area ratio per visual field was 15.32 ± 1.15% and 9.70 ± 1.12%, respectively. However, there was no apparent difference between the two groups (p> 0.05). On the other hand, the GelMA-AFN group showed obviously higher expression levels of NF and LN compared to the other two groups. The NF positive area ratio per visual field was 43.72 ± 1.81% in the GelMA-AFN group, which was 3.01 times higher than that of the SCI group and 2.85 times higher than that of the GelMA group. Similarly, the LN positive area ratio per visual field in the GelMA-AFN group was 29.63 ± 2.53%, which was 3.23 times higher than that of the SCI group and 3.05 times higher than that of the GelMA group (p < 0.01, Figure 9E, F). These findings indicate that GelMA-AFN effectively enhances the content of LN, a nerve growth substrate, within the injured spinal cord.

### Activation of functional neurons, axonal remyelination, and synapse formation in lesion area by GelMA-AFN transplantation following SCI

**Figure 10:**
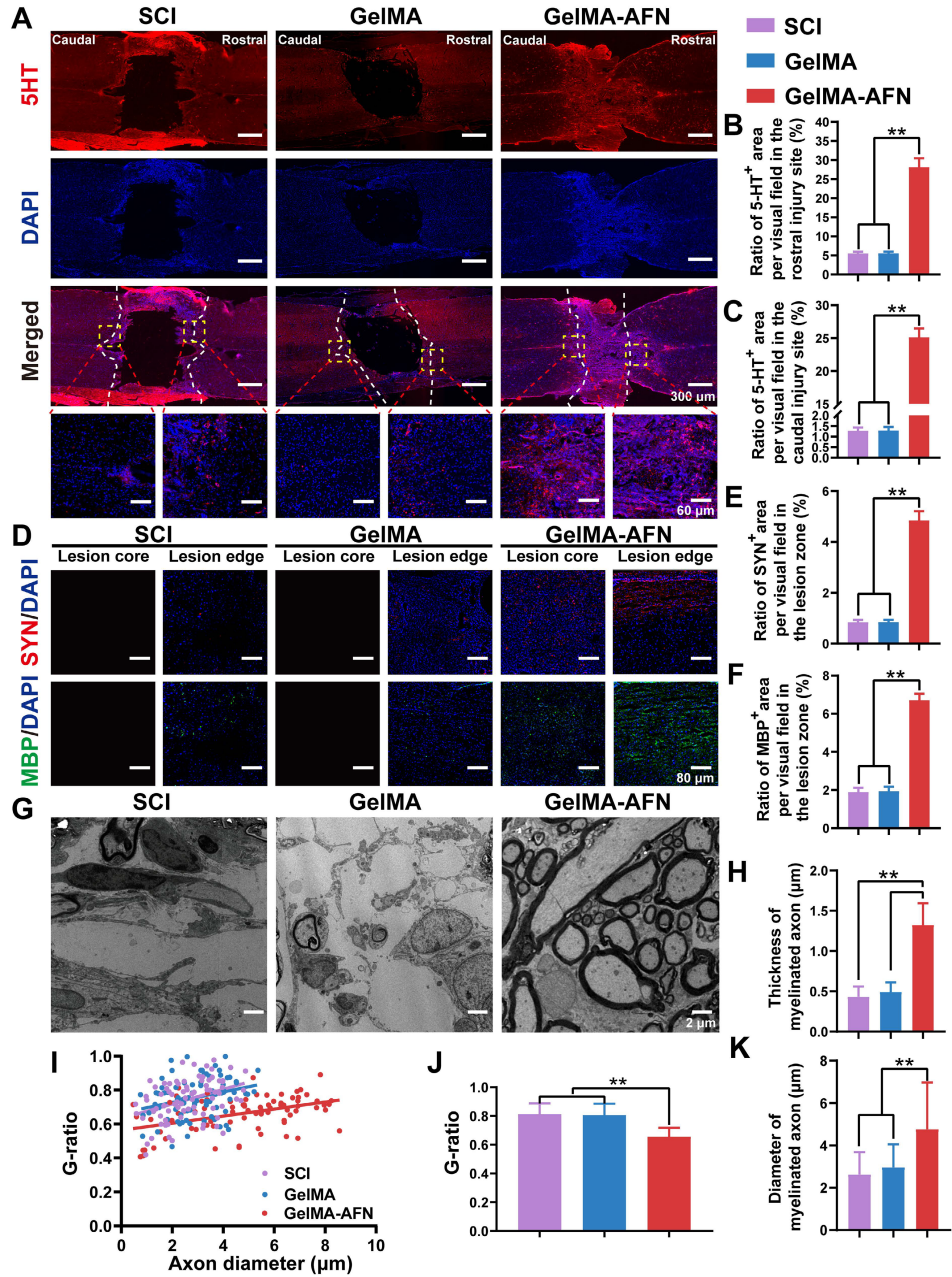
** Activation of functional neurons, Axonal remyelination, and synapse formation in lesion area by GelMA-AFN transplantation 8 weeks after SCI. (A)** Representative immunofluorescence images of the injury site. The spinal cords were stained with anti-5-hydroxy tryptamine (in red) and DAPI (in blue, marker of nuclear) (scale bar: 300 µm, 60 µm). **(B, C)** The mean ratio of 5-HT^+^ fluorescence area per visual field in the rostral and caudal injury site. **(D)** Representative immunofluorescence images of injury site. The spinal cords were stained with anti-SYN (in red, marker of synapse), anti-MBP (in green, marker of myelin sheath), and DAPI (in blue, marker of nuclear) (scale bar: 80 µm). **(E, F)** Mean ratio of fluorescence positive area per visual field at the injury site. **(E)** SYN, **(F)** MBP. **(G)** Transmission electron micrographs of regenerated axons and myelin sheath in the middle portion at 8 w post-injury. **(H)** Thickness of myelinated axon (µm). **(I)** Scatter plot showing g-ratio and axon diameter for all groups at 8 w post-injury. **(J)** Quantification of the g-ratio. **(K)** Diameter of myelinated axon (µm) (**p < 0.01, *n* = 6).

Subsequently, we marked the serotonergic axons with anti-5-hydroxytryptamine (5-HT) in the sections of spinal cord. We found that the trends of 5-HT results were similar to those of Tuj1 staining. No apparent distinction was found in the rostral region of the spinal cord between the SCI (5.49 ± 0.47%) and GelMA group (5.50 ± 0.48%) (p > 0.05). In contrast, the rats in the GelMA-AFN group (28.11 ± 2.38%) had more 5-hydroxytryptaminergic positive area ratio per visual field in the rostral region of the spinal cord than other groups, which was 5.12 folds than that of the SCI group and 5.11 folds than that of the GelMA group (p < 0.01, Figure 10A, B). In the caudal region of the spinal cord, a lower 5-HT^+^ area ratio per visual field was observed in the SCI (1.28 ± 0.15%) and the GelMA (1.29 ± 0.17%) groups. Conversely, the caudal region of the spinal cord in the GelMA-AFN group (25.13 ± 1.34%) contained a similar area ratio of 5-HT^+^ to the rostral region, which was 19.63 times that of the SCI group and 19.48 times greater than the GelMA group (p < 0.01, Figure 10A, C). The results suggest that after GelMA-AFN treatment, 5-HT can pass through the lesion of the spinal cord. The microenvironment of SCI may affect the synthesis and secretion of 5 HT and affect the activity of functional neurons.

Eight weeks post-SCI, immunostaining for synaptophysin (Syn) was performed to evaluate synapse formation. As depicted in Figure 10D, a intensive region of Syn^+^ signal was observed in the injured area of the GelMA-AFN group rats. Conversely, the Syn^+^ area ratio per visual field was rarely lower at the lesion margins in the other groups, where a necrotic void was present at the center with no positive signal. Synapse formation was significantly improved in the lesion area treated with GelMA-AFN (4.84 ± 0.37%), with a positive area ratio per visual field being 5.76 times higher than in the SCI group (0.84 ± 0.09%) and 5.69 times higher than in the GelMA group (0.85 ± 0.09%) (p < 0.01, Figure 10D, E). To assess remyelination of the regenerated axons, we performed staining for the myelin basic protein marker (MBP) in three groups of rats. The results showed there was evident remyelination in the lesion core and edge area in the GelMA-AFN group. At the lesion margins, MBP positive area ratio per visual field was slightly lower in both the SCI and GelMA groups (Figure 10D). In these groups, there was a central necrotic void with no positive cells. Remyelination was significantly increased in the lesion area treated with GelMA-AFN (6.71 ± 0.34%), with MBP positive area ratio per visual field in the lesion site being 3.53 folds than the SCI group (1.90 ± 0.22%) and 3.46 times higher than the GelMA group (1.94 ± 0.24%) (p < 0.01, Figure 10D, F). These results demonstrated that GelMA-AFN not only enhances axon growth at the lesion site but also promotes synapse formation along both proximal and distal axons. Furthermore, these newly formed axons are enveloped by emerging myelin sheath, which aids in the accurate and swift transmission of electrical signals.

Remyelination plays a crucial role in neural signaling involved in functional recovery after SCI. Eight weeks after SCI, regenerative axons and myelination in the injured area were examined using transmission electron microscopy (Figure 10G). The average thickness of myelinated axons in the GelMA-AFN group was 1.32 ± 0.27 μm, which was 2.69 folds than that of the GelMA group (0.49 ± 0.12 μm) and 3.07 times that of the SCI group (0.43 ± 0.13 μm) (p < 0.01, Figure 10H). SCI leads to a reduction in both axon and myelin area, resulting in a global contraction of nerve fibers. For another thing, GelMA-AFN appears to have neuroprotection by preserving the structure of nerve fibers, preventing axonal contraction and myelin sheath thinning. Electron microscopy results reflected that the majority of regenerated neurofilaments in the GelMA-AFN group were tightly ensheathed by myelin, with an average G ratio (axon diameter/axon + myelin diameter) of 0.66 ± 0.11. However, both the SCI group (0.75 ± 0.12) and the GelMA group (0.76 ± 0.13) exhibited less remyelination, and in some visual fields, axons were completely unmyelinated (Figure 10I, J). This suggests that SCI leads to spontaneous remyelination, but with lower efficiency and thinner remyelination, while GelMA-AFN creates an advantageous microenvironment for remyelination at the lesion. Furthermore, the results demonstrated that the axon diameter in the GelMA-AFN group was significantly larger than in the other two groups. The average diameter of myelinated axons in the GelMA-AFN group was 4.76 ± 2.21 μm, which was 1.61 times that of the GelMA group (2.96 ± 1.09 μm) and 1.82 times that of the SCI group (2.62 ± 1.07 μm) (p < 0.01, Figure 10K). These results indicated that GelMA-AFN promoted axon regeneration, increases axon diameter, and promotes functional nerve repair following SCI.

### Gene expression changes induced by GelMA-AFN in SCI tissue revealed by RNA sequencing at 8 weeks post-SCI

**Figure 11:**
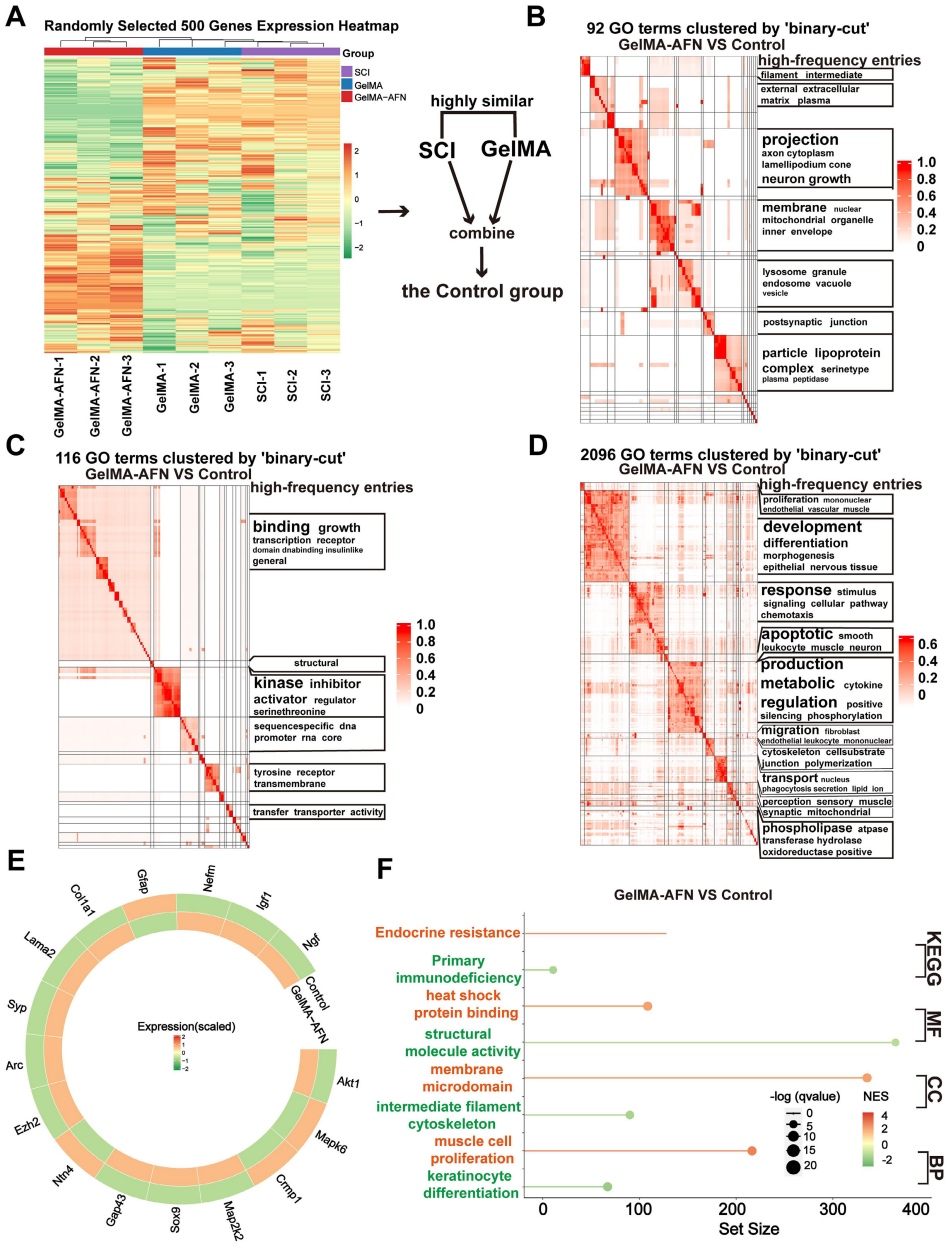
** Gene Expression Changes Induced by GelMA-AFN in spinal cord tissue 8 weeks post-SCI. (A)** Clustering heat map based on randomly selected 500 gene expressions. **(B)** Gene Ontology (GO) enrichment analysis of DEGs focused on Cell Component (CC). **(C)** GO enrichment analysis of DEGs focused on Biological Process (BP). **(D)** GO enrichment analysis of DEGs focused on Molecular Function (MF). **(E)** Circos heat map of 16 DEGs expression associated with nerve regeneration. **(F)** The results of GSEA enrichment analysis between the GelMA-AFN and control groups show the top pathway with maximum and smallest NES (enrichment score) values in each category based on the GO (CC, MF, BP) and KEGG database. (|log2FC| > 1.5 and p < 0.05, *n* = 6).

To investigate gene expression changes at a later stage, total RNA was extracted from the central spinal cord tissues 8 weeks post-injury for RNA sequencing. DEGs were identified based on |log2FC| > 1.5 and p-value < 0.05. Clustering heatmap analysis revealed no significant differences in gene expression between the SCI and GelMA groups, prompting their combination into a Control group for comparison with the GelMA-AFN group (Figure 11A). Functional pathway enrichment analysis using the Gene Ontology (GO) database showed that DEGs were predominantly enriched in pathways related to nerve regeneration, axonal growth, synapse formation, myelin sheath formation, ECM synthesis, and membrane potential regulation (Figure 11B, C, D). GESA revealed that the expression of neural repair pathway was obviously elevated in the GelMA-AFN group compared to the Control group. A total of 783 DEGs were evaluated, incorporating 376 downregulated and 409 upregulated genes. Genes associated with promoting neural repair, such as Ngf, Igf1, Ntn4, and Ezh2, were upregulated in the GelMA-AFN group, leading to an improved spinal cord microenvironment, increased neuronal survival ratios, and enhanced axonal growth. Upregulation of Crmp1 and Nefm supported neuronal remodeling and axonal cytoskeleton integrity, while upregulation of Arc and Syp facilitated calcium ion transport and synaptic formation. Activation of MAPK and MAP2K2 genes promoted the connection between neuronal axons and synapses, further supporting neural circuit reconstruction. Additionally, upregulation of Col1A1 and Gap43 genes resulted in increased ECM synthesis, promoting axonal regeneration. Conversely, genes detrimental to nerve repair, such as GFAP and SOX9, were significantly downregulated in the GelMA-AFN group, indicating that glial scar proliferation was inhibited and the environment for nerve growth was improved (Figure 11E, F, Figure S4). In order to confirm the precision of the RNA transcriptome findings, we selected PCR to assess the expression of NGF, Igf1, and other genes in spinal cord tissue 8 weeks post-injury. The results were consistent with the RNA Sequencing (Figure S2B).

### Electrophysiological and electromyographic improvements following GelMA-AFN transplantation after SCI

**Figure 12:**
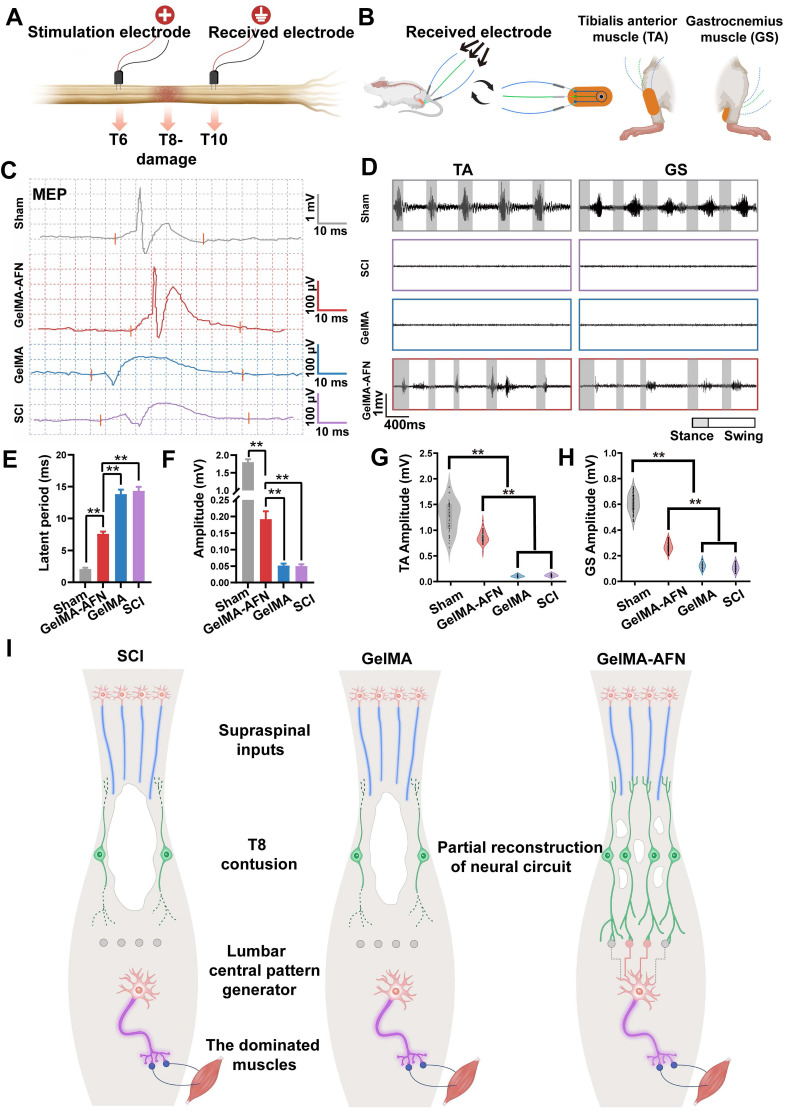
** Electrophysiological and electromyographic improvements following GelMA-AFN transplantation 8 weeks after SCI. (A)** The detection of motor evoked potential (MEP). **(B)** The detection of electromyography of gastrocnemius (GS) and tibialis anterior muscle (TA). **(C)** Representative images of stimulating potential in rats with different treatments. **(D)** Representative images of GS and TA potentials in rats with different treatments. **(E, F)** Latent period (ms) and amplitude (mV) of MEP. **(G, H)** TA and GS amplitude (mV). **(I)** Neural circuit reconstruction in different groups of rats at 8 weeks (p < 0.01, *n* = 6).

Electrophysiological assessments were performed 8 weeks post-SCI to examine how functional recovery correlates with axonal regeneration in the three experimental rat groups. Stimulation electrodes were placed dorsally at T6, two segments above the injured site, and recording electrodes were positioned at T10, two segments below the damaged area (Figure 12A). In the Sham group, signal transmission exhibited a significantly shorter latency (2.84 ± 0.32 ms) compared to both the SCI group (13.26 ± 0.89 ms) and the GelMA group (12.87 ± 0.85 ms). The GelMA-AFN group exhibited marked improvement in electrical conduction (8.67 ± 0.24 ms), though the latency remained longer than that of the Sham group (p < 0.01, Figure 12C, E). Regarding evoked potential amplitude, the GelMA-AFN group (0.19 ± 0.03 mV) showed substantial recovery compared to the SCI group (0.050 ± 0.006 mV) and the GelMA group (0.052 ± 0.006 mV), though it did not reach the level observed in the Sham group (1.80 ± 0.25 mV). The amplitude in the GelMA-AFN group was 3.8 folds than the SCI group and 3.7 folds than the GelMA group (p < 0.01, Figure 12C, F), suggesting that GelMA-AFN may function as a "relay," facilitating the repair of impaired neural pathways and allowing the conduction of descending signals through the injured interface.

Eight weeks post-SCI, electromyography (EMG) recordings were obtained to evaluate hindlimb muscle activity during free walking. Ankle flexor (tibialis anterior, TA) and extensor (gastrocnemius soleus, GS) muscles in the SCI and GelMA group showed minimal activation, with sporadic signals in other treatment groups. In contrast, GelMA-AFN-treated rats exhibited consistent activation of the TA and GS during various steps (Figure 12B, D). Statistical analysis of the EMG data revealed more regular TA and GS rhythms in the GelMA-AFN group, with scatter plots showing a wider and more concentrated distribution around the central (zero) position, resembling the pattern observed in the Sham group (Figure 12G, H). This suggests that GelMA-AFN treatment significantly improves muscle control and coordination. The mean TA amplitude in the GelMA-AFN group (0.71 ± 0.12 mV) was 5.47-fold higher than in the GelMA group (0.13 ± 0.02 mV) and 5.92-fold higher than in the SCI group (0.12 ± 0.03 mV). Similarly, the mean GS amplitude in the GelMA-AFN group (0.28 ± 0.05 mV) was 2.8-fold than in the GelMA group (0.10 ± 0.01 mV) and 3.1-fold than in the SCI group (0.09 ± 0.01 mV). The maximum TA and GS amplitudes in the GelMA-AFN group recovered to 56% and 46% of the Sham group values (1.26 ± 0.27 mV and 0.61 ± 0.08 mV, respectively) (p < 0.01). The findings suggest that GelMA-AFN treatment facilitate efficient transmission of neural signals to innervated muscles across the SCI site, demonstrating significant reconstruction of the neural circuitry (Figure 12I).

### GelMA-AFN improves motor function and attenuates skeletal muscle atrophy in rats following SCI

**Figure 13:**
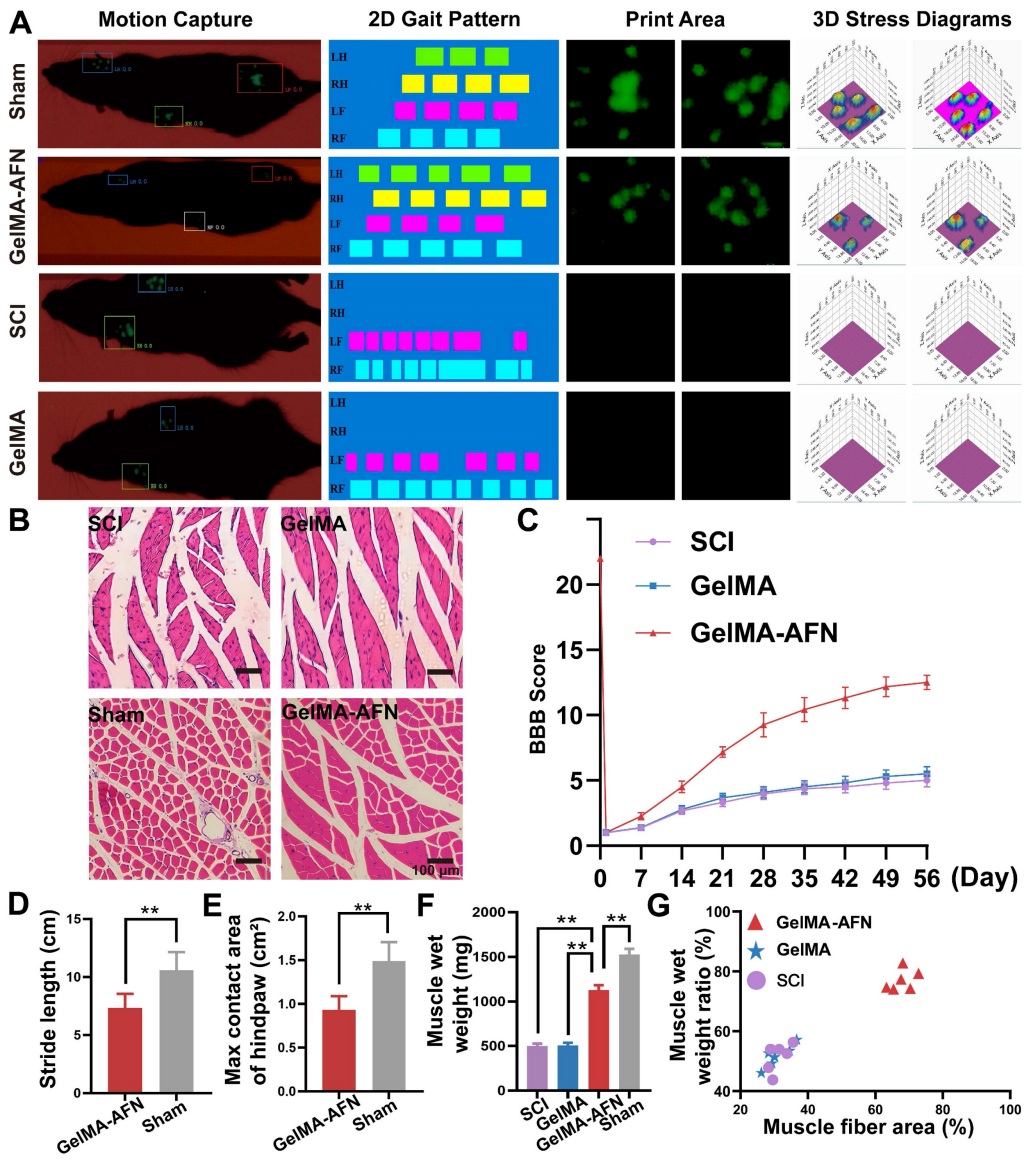
** GelMA-AFN improves motor function and attenuates skeletal muscle atrophy in rats 8 weeks after SCI. (A)** Unprocessed control charts obtained using the Green walk software including gait patterns, representative images showing the maximum print area, and print and gait patterns for the left (LH) and right (RH) hind paws (LF = left front paw; RF = right front paw; LH = left hind paw; RH = right hind paw). **(B)** Transsection of gastrocnemius muscle H&E staining from different groups of rats. (scale bar: 100 µm) **(C)** BBB scores. **(D)** Maximum hind paw contact area. **(E)** The stride length of rats. **(F)** Mean gastrocnemius muscle wet weight (mg). **(G)** gastrocnemius muscle wet weight ratio and fiber area. X-axis: The ratio of muscle fiber area in different samples from three groups to the mean fiber area of the Sham group (%). Y-axis: The ratio of gastrocnemius muscle wet weight in different samples from three groups to the mean wet weight of the Sham group (%). (p < 0.01, *n* = 6).

Behavioral assessments are vital for assessed potential treatments for SCI in preclinical trials. In order to obtain comprehensive and quantitative evaluation of motor function, we used a small animal gait system. At 8 weeks post-surgery, this system enabled a comprehensive analysis of rat locomotion using both 2D and 3D stress maps (Figure 13A). At this time, the hind limbs of both SCI and GelMA rats remained paralyzed and showed no signs of stress. Therefore, the gait analysis results for these two groups only included data from the forelimbs. However, the GelMA-AFN group displayed slow movement with reduced support strength. The rear paw contact area (0.932 ± 0.156 cm^2^) in this group remained smaller than that of the Sham group (1.487 ± 0.218 cm^2^), and the rear paw stayed on the ground for a longer duration (p < 0.01, Figure 13E). These findings indicate that while GelMA-AFN improves hind limb strength in rats, it is insufficient for full-body support. Some data can be visualized in a 2D map. Additionally, significant differences were displayed in step length among the four groups. Both the SCI group and GelMA group were unable to stand, while the average step length of the GelMA-AFN group was 7.35 ± 1.21 cm, accounting for 69.47% of the Sham group's step length (10.58 ± 1.56 cm) (p < 0.01, Figure 13D). The BBB scores for the three groups from weeks 1-8 corresponded to the results of the gait analysis. At the eighth week, the SCI group and the GelMA group still scored less than 5 points, whereas the GelMA-AFN rats achieved a score of 12 points due to improved mobility, although their coordination remained insufficient. These findings indicate that although GelMA-AFN effectively enhances the locomotor ability of SCI rats (p < 0.01, Figure 13C), it falls short of complete functional recovery.

Skeletal muscle atrophy was assessed 8 weeks post-SCI. Compared to the Sham group, SCI induced varying degrees of fiber atrophy. H&E staining of transverse muscle sections showed significant atrophy in both the SCI group and GelMA group (Figure 13B). The ratio of muscle fibers was measured to be 43.56 ± 3.96 % and 43.79 ± 3.20 %, respectively 50. The GelMA-AFN group had a higher ratio of muscle fiber area (65.31 ± 2.95%), which was smaller than the Sham group (82.58 ± 2.24%). The ratio of muscle fiber area in the GelMA-AFN group was 0.77 times than that of the Sham group, but distinctly higher than the SCI group (1.50 times) and GelMA group (1.49 times) (p < 0.01, Figure 13G). Additionally, the GelMA-AFN group (1129.68 ± 52.5 mg) showed improvement in wet weight of gastrocnemius muscle compared to the SCI (498.72 ± 29.21 mg) and GelMA groups (505.33 ± 30.19 mg) (p < 0.01, Figure 13F). These results suggest that GelMA-AFN effectively improves muscle fiber atrophy caused by SCI. Gastrocnemius muscle wet weight in the SCI, GelMA, and GelMA-AFN groups was 0.31, 0.30, and 0.68 times than the Sham group (p < 0.01, Figure 13G). GelMA-AFN may have facilitated the reconstruction of the motor nerve loop in the rat spinal cord, resulting early recovery of the rat hind limb and preventing severe gastrocnemius atrophy. However, the weight of GelMA-AFN did not fully restore muscle function in rats compared to the Sham group.

## Discussion

The pathological processes of SCI are highly complex, involving ischemia, hypoxia, and free-radical formation, which subsequently lead to inflammation, astrocyte proliferation, neuronal death, and persistent neurological dysfunction 51. Studies suggest that activating neuronal regeneration and improving the early inflammatory environment can partially ameliorate SCI outcomes 52, 53. However, due to limitations in current drug delivery systems, most previous research has focused on either improving the early inflammatory environment or enhancing neuronal regeneration, leading to limited improvements in SCI prognosis. In response, we conceptualized a hydrogel microsphere design with an outer layer for early degradation and anti-inflammatory drug delivery and an inner layer for sustained release of neurotrophic agents. This design aims to suppress inflammation during the acute phase and promote neural repair during the recovery phase.

Our findings indicate that GelMA is suitable as an outer-layer drug carrier for SCI treatment; however, its mechanical strength and elasticity are insufficient for long-term drug release *in vivo*. Incorporating nanoclay significantly strengthened the cross-linked structure and extended the degradation time. Previous studies have shown that the addition of 1.5% nanoclay to 5% GelMA increases viscosity, enhances shear-thinning properties, and improves printability and shape fidelity 54. Incorporating nanoclay into GelMA hydrogels also facilitates the sustained release of exosomes 55. Our study demonstrated that incorporating nanoclay into GelMA extended the degradation time to over one month, supporting our objective of long-term drug release. To address inflammation and low neuronal regeneration post-SCI, we selected drugs with both anti-inflammatory and neurogenic properties. ANXA1 was chosen for the outer microsphere layer due to its ability to inhibit neutrophil aggregation, induce M2 macrophage polarization, and suppress inflammatory responses. NGF was chosen for the inner microsphere layer due to its well-established role in promoting the growth, differentiation, and maturation of neurons in both the central and peripheral nervous systems, thereby accelerating post-injury repair. Additionally, given the importance of ECM components for axonal growth, we included FN in the inner microsphere layer. FN is a high-molecular-weight glycoprotein that forms a network-like structure in the ECM, providing the physical support necessary for axonal guidance and growth. Previous studies have shown that fibronectin secreted by juvenile microglia rapidly forms ECM bridges after SCI, promoting the reconnection of injury sites and facilitating axonal extension. A major challenge lies in integrating these components while ensuring that the microsphere structure aligns with the biomimetic therapeutic principles needed to address SCI pathology, maintaining uniform size and shape 13. To overcome this, we developed a microfluidic chip specifically designed for the one-step fabrication of GelMA core-shell microspheres. By leveraging adhesion and shear forces between different fluids, we successfully produced biomimetic dual-layer multifunctional microspheres. Material characterization confirmed that our microspheres exhibit distinct layering, uniform size and morphology, and appropriate degradation times. Notably, nanoclay played a critical role in extending the degradation time of GelMA. The GelMA-AFN microspheres remained intact after 30 days. The strong electrostatic interactions between GelMA's positively charged chains and nanoclay's negatively charged chains significantly enhanced crosslinking and mechanical strength, consistent with previous findings by Hu *et al.* and Adib *et al.* Adib's research also demonstrated that GelMA-nanoclay scaffolds retained their shape and mechanical properties for up to 21 days, further confirming nanoclay's capacity to improve hydrogel physical properties 38, 56. Our experiments revealed that the swelling ratio of GelMA-AFN microspheres at any given time point was lower than that of pure GelMA microspheres without nanoclay. This indicates that the strong electrostatic interactions between GelMA and nanoclay effectively reduced water absorption and swelling, contributing to the structural stability of the microspheres. Furthermore, no premature release of inner-layer drugs was detected while the outer layer remained intact, further supporting the robustness of the cross-linked structure. Interestingly, we observed that a 2% nanoclay ionic solution forms a gel after standing at room temperature for several hours. According to Kim *et al.*, nanoclay interacts with solution ions through edge-to-face interactions, leading to gel formation 57. We hypothesize that under suitable conditions, nanoclay in the microspheres could interact with the surrounding solution to form secondary gels, further prolonging the inner layer's degradation time. Recent studies, such as Ji *et al.*'s development of a nanoclay hydrogel vaccine for colon cancer in mice, and Huang *et al.*'s targeted delivery system for treating thyroid cancer in mice, demonstrate the safety and reliability of nanoclay in biological systems 58, 59. Our findings also showed accelerated microsphere degradation between days 14 and 28, likely due to excessive swelling disrupting the tight cross-linked structure. However, degradation slowed between days 28 and 35, potentially due to secondary gel formation from nanoclay interactions, as observed in Kim *et al.*'s study and in our experiments.

The potent anti-inflammatory effects of ANXA1 were validated in our study, although its application in spinal cord inflammation has rarely been explored. Within 1-3 days post-SCI, neutrophils rapidly accumulate at the injury site, disrupting the blood-spinal cord barrier and triggering a cascade of inflammatory responses 60. Our findings demonstrate that ANXA1 effectively inhibits neutrophil aggregation in the injured spinal cord, consistent with the findings of Ansari *et al.* 13, who reported that ANXA1 regulates activated neutrophils through the Akt and ERK1/2 pathways. By interacting with FPR2 (formyl peptide receptor 2)/ALX (lipoxin A4 receptor) on neutrophils, ANXA1 promotes their apoptosis, thereby aiding in the resolution of inflammation. Reports also suggest that ANXA1 enhances M2 macrophage polarization in certain tissues, a result we also observed in the spinal cord, potentially linked to the activation of the AMPK pathway 14, 16. Moreover, ANXA1 reduced the overall macrophage count at the injury site, a finding that has not been extensively reported, warranting further investigation into the underlying mechanisms. Support for this is provided by Ferraro *et al.*, who observed a significant reduction in myocardial macrophage infiltration following myocardial infarction in mice treated with ANXA1 61.

The anti-inflammatory mechanisms of AnxA1 primarily involve its interaction with formyl peptide receptors (FPRs), which inhibit cell adhesion and migration. Glucocorticoids (GCs) upregulate AnxA1 expression and release, promoting rapid phosphorylation via protein kinase C (PKC) activation and subsequent membrane translocation 62. At the cell surface, AnxA1 binds to phosphatidylserine (PS), mediating apoptotic body phagocytosis. Additionally, AnxA1 suppresses cPLA2 (cytosolic phospholipase), prostaglandins, and COX-2 (cyclooxygenase), while inhibiting NF-κB activation through binding 63. Glucocorticoid receptor (GR) dimerization and nuclear translocation facilitate transcriptional regulation by binding to glucocorticoid response elements (GREs). Following SCI, peripheral macrophages are recruited to the injury site, where they secrete pro-inflammatory cytokines and complicate distinctions between macrophage and microglial roles in shaping the microenvironment 64. In the acute phase of SCI, microglia predominate, constituting approximately 81% of cells at the lesion edge and 89% at the lesion center, with fewer blood-derived macrophages 65. While IL-18 is expressed in myeloid cells of SCI patients, IL-1β and IL-10 are undetectable, suggesting that microglia are the primary source of these cytokines during the acute phase. This underscores the ability of GelMA-AFN to modulate inflammation by suppressing microglial activation and limiting infiltration during the acute phase.

Rats in the GelMA-AFN group exhibited significantly less glial scarring compared to the other groups, supporting findings by Ma *et al.* 66 that early anti-inflammatory intervention reduces reactive astrocyte proliferation and glial scar formation after SCI. Microglia play a key role in triggering astrocyte proliferation, and anti-inflammatory treatment downregulates the p38 MAPK and Erk1/2 pathways, suppressing neuroinflammation. This inhibits astrocyte differentiation into the A1 subtype via the NF-κB pathway, ultimately reducing glial scar formation 67. These findings suggest that ANXA1 alleviates acute tissue damage and neuronal loss following SCI, improving conditions for effective treatment 68. Post-SCI, the lesion area often contains cavities that hinder axonal growth. While axons can overcome glial scar barriers, they cannot traverse the cavity to establish synaptic connections. Research shows that cavity repair in mouse SCI relies on cells secreting fibronectin (FN) to form an ECM bridge 69. In adult rats, the lesion area lacks sufficient ECM, necessitating the introduction of exogenous FN to promote axonal extension. In our study, the GelMA-AFN group showed only 11.30% of the cavity area in the SCI group and 12.35% of that in the GelMA group, with extensive axonal growth observed, likely due to the sustained release of FN from GelMA-AFN microspheres, which filled the cavity and supported axonal extension.

Immunofluorescence staining revealed that nerve fibers co-localized with FN, laminin, and collagen I in the lesion area, confirming this process. The widespread axonal growth in the lesion area suggests the possibility of successful electrical signal transmission across the SCI site 70. Electrophysiological assessments indicated that the GelMA-AFN group successfully transmitted external electrical stimulation from the proximal to the distal end of the injury site, whereas such conduction was nearly absent in the SCI and GelMA groups. This suggests that GelMA-AFN microspheres sustained the release of NGF, promoting synapse formation and neural circuit reconstruction in the spinal cord, enabling proximal electrical signals to reach distal targets. Although the evoked potential waveform in the GelMA-AFN group closely resembled that of normal rats, the maximum amplitude remained notably lower than that in healthy controls, consistent with findings by Zuo *et al.* 71. Previous studies have suggested that even after the reconstruction of synapses and neural circuits post-SCI, functional recovery is not guaranteed. Reconstructed circuits may require extensive retraining to gradually regain full functionality 72.

RNA sequencing results further validated our experimental findings at the gene expression level. Functional enrichment analysis effectively demonstrated that GelMA-AFN regulates key processes within the spinal cord inflammatory microenvironment at different stages post-SCI. At 7 days post-injury, GelMA-AFN modulates processes related to apoptosis, ion transport, cytokine secretion, macrophage chemotaxis, and inflammasome formation 53. At 7 days post-SCI, GelMA-AFN was found to regulate apoptosis-related proteins such as BAX and BCL2, downregulate pro-inflammatory cytokines like IL-1β, upregulate anti-inflammatory cytokines like IL-10, and modulate the PI3K/AKT pathway, thereby inhibiting apoptosis and reducing early neuronal loss. At 8 weeks post-injury, GelMA-AFN influences pathways involved in axonal growth, synapse formation, neurotransmitter transmission, growth factor synthesis and secretion, and cell adhesion. Additionally, protein-protein interaction network analysis identified critical molecular players closely associated with spinal cord repair. At 8 weeks post-SCI, GelMA-AFN upregulated the expression of growth factor genes such as Igf1 and Ngf, as well as ECM genes, including Col1a1 and Lama2. GelMA-AFN also upregulated SOX9, a key regulator of neural stem cell maintenance and differentiation, while modulating the RAF-MAP2K pathway to promote axonal regeneration and functional remodeling 73.

To assess hindlimb motor function recovery after SCI in rats, we used BBB scoring and gait analysis. Behavioral assessments showed that rats in the GelMA-AFN group exhibited significant improvements in hindlimb motor ability, including the ability to support their body weight and move slowly. Notably, GelMA-AFN microspheres induced greater motor function improvement than treatments focusing solely on early inflammation control. These findings align with those of Moretti *et al.*, who demonstrated that multi-targeted therapeutic approaches lead to superior behavioral outcomes compared to single-target treatments in SCI models 74. In the assessment of muscle atrophy, the GelMA-AFN group exhibited higher gastrocnemius muscle wet weight and muscle fiber area, with muscle morphology closely resembling that of healthy rats. The muscle fiber area in the GelMA-AFN group was significantly larger than in both the SCI and GelMA groups. These results suggest that GelMA-AFN facilitated early neural circuit reconstruction in the spinal cord regions controlling hindlimb movement, allowing for partial functional recovery and preventing severe skeletal muscle atrophy 75.

Integrating transplanted microspheres with host tissue is critical for restoring neural function. In this study, we first demonstrated the neuroprotective effects of GelMA-AFN in diverse environments, supporting neuronal survival. Reestablishing synaptic connections and neural networks in the injured spinal cord is crucial for functional recovery. We observed substantial new synaptic connections between regenerating neurons at the injury/graft site and regenerated host neural fibers. We propose that four key factors contributed to the restoration of neural connections at the injury site: (1) Early anti-inflammatory intervention reduced neuronal loss and improved the microenvironment for neuron survival. (2) Sustained NGF release created a pro-regenerative microenvironment that promoted axonal growth. Exogenous NGF not only supported neuronal differentiation and survival but also potentially enhanced the regeneration of descending motor tracts and ascending sensory tracts 76. (3) The incorporation of FN in GelMA-AFN facilitated tissue migration and the growth of regenerating neural fibers 77. The regulation of permissive ECM proteins, such as laminin, likely counteracted the inhibitory effects of chondroitin sulfate proteoglycans, the primary component of glial scars, on axonal regeneration. Studies suggest that FN molecules provide substrates for regenerating tissue while remaining neurons offer targets for newly growing neural fibers 78. (4) The protein factors released from GelMA-AFN effectively repaired the “neural relay” at the injury site. In this scenario, endogenous neurons at the injury site can transmit neural signals, and when they establish contact with relay neurons, long-distance regeneration of descending motor tracts becomes feasible 72. Previous studies have reported that descending 5-HT fibers of the raphe-spinal tract (RpST) are closely associated with motor recovery, highlighting the importance of neurotrophic factors 45. Our results indicate that regenerated and existing neural fibers formed connections, allowing electrical signals to transmit smoothly through the injury site. Brain-derived signals were relayed from the spinal cord to the hindlimbs, significantly improving motor function in the rats. Therefore, our strategy produced a dual-layer multifunctional composite microsphere that successfully restored both ascending and descending neural signals, aiding in the repair of sensory and motor pathways.

## Conclusion

In summary, we developed a novel microfluidic chip and engineered a dual-layer composite GelMA microsphere tailored to match the dynamic pathological changes following SCI. These microspheres exhibit uniform size, well-defined layering, ideal degradation timing, targeted drug loading, and no cytotoxicity. Our experiments demonstrated that these microspheres effectively modulate the early inflammatory environment post-SCI while providing sustained release of therapeutic agents during the mid-to-late stages of injury, thereby promoting neural regeneration and myelination. This significantly improved motor function in rats. Collectively, our findings indicate that GelMA-AFN microspheres offer a multifaceted approach to treating SCI and hold promise for future clinical applications.

## Supplementary Material

Supplementary figures.

## Figures and Tables

**Scheme 1:**
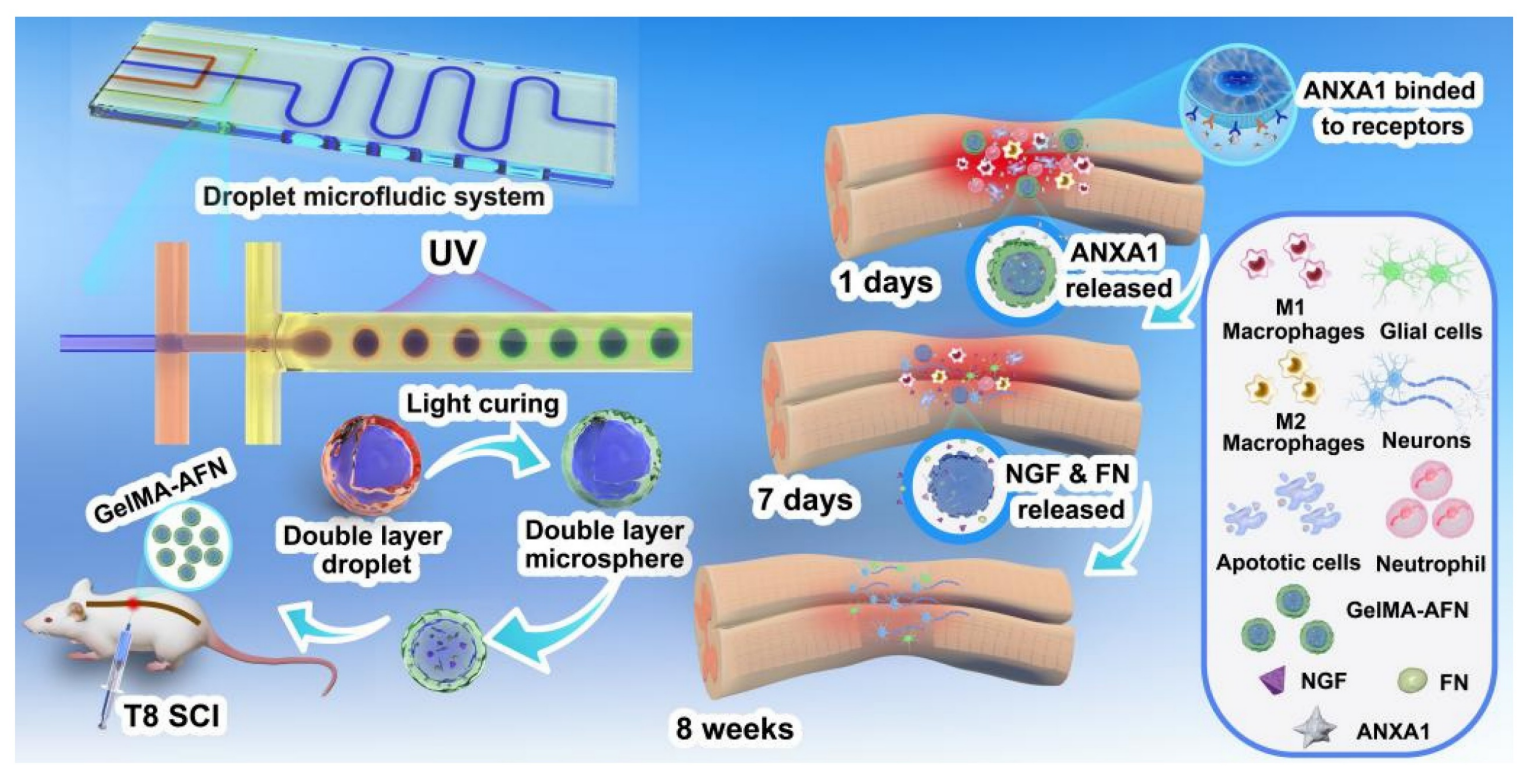
** UV-crosslinked microfluidic chip for GelMA-AFN preparation and the associated *in vivo* biological mechanism**.
