# Supplementary figures and images for: Biomimetic core-shell GelMA microspheres co-delivering ANXA1, NGF, and fibronectin enable phase-matched immunomodulation and neurorepair after spinal cord injury
Source: Theranostics. 2026 Jan 1;16(1):483–515. doi: 10.7150/thno.120426 (PMC12665142; doi:10.7150/thno.120426)

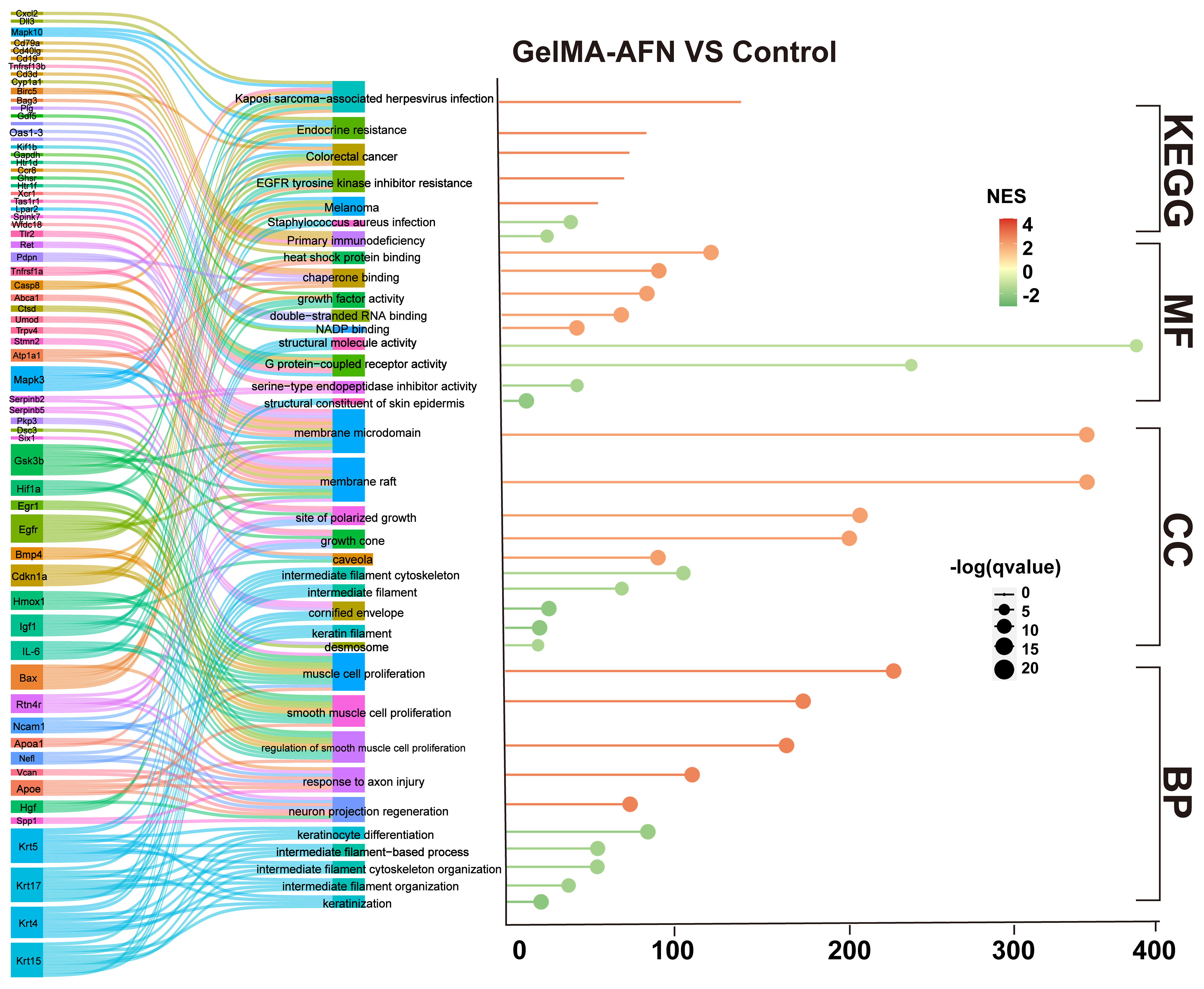

Supplement: Supplementary file 1 — Supplementary figures. [file thnov16p0483s1.zip › Figure SS4.tif]

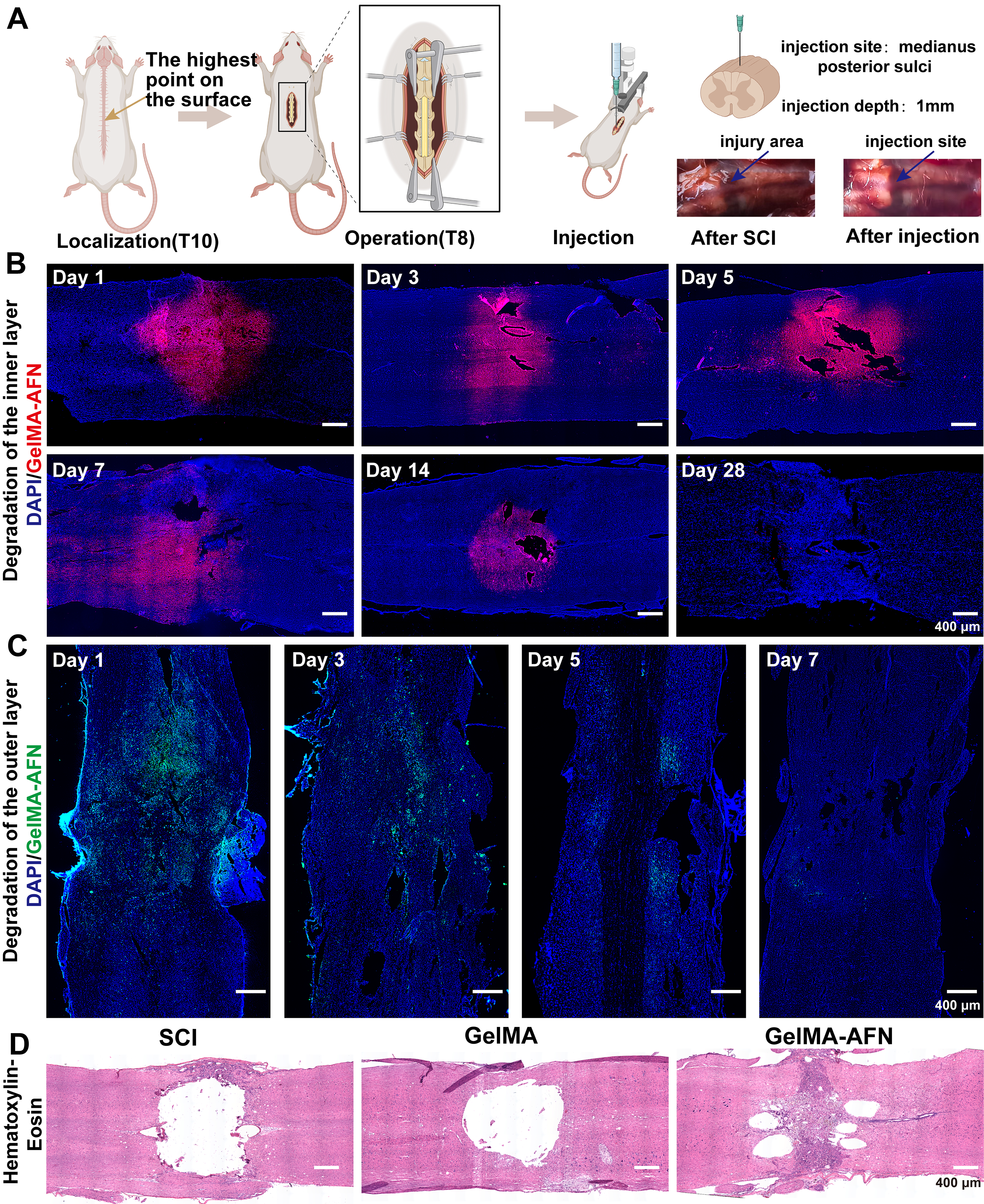

Supplement: Supplementary file 1 — Supplementary figures. [file thnov16p0483s1.zip › Figure S1.tif]

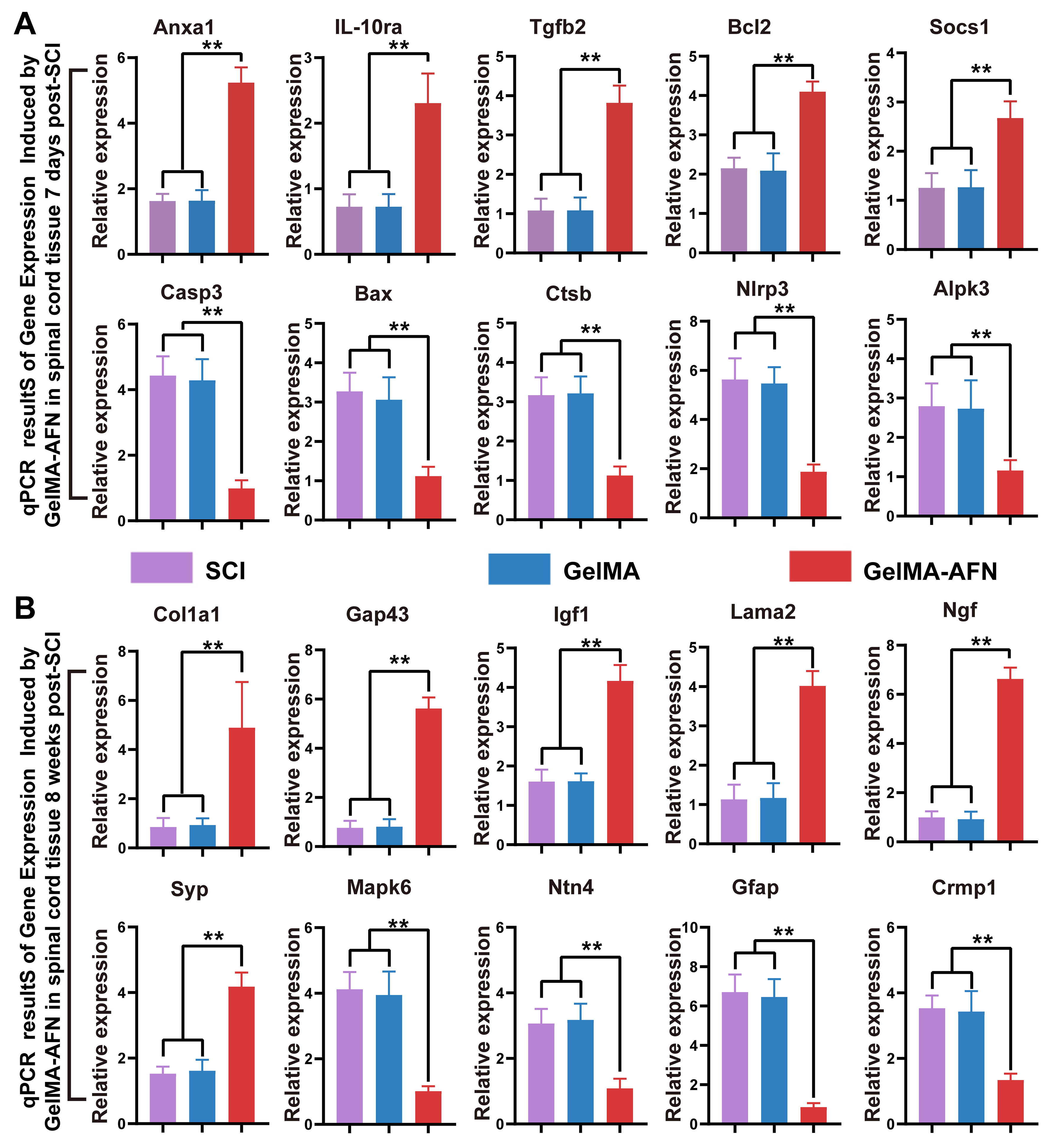

Supplement: Supplementary file 1 — Supplementary figures. [file thnov16p0483s1.zip › Figure S2.tif]

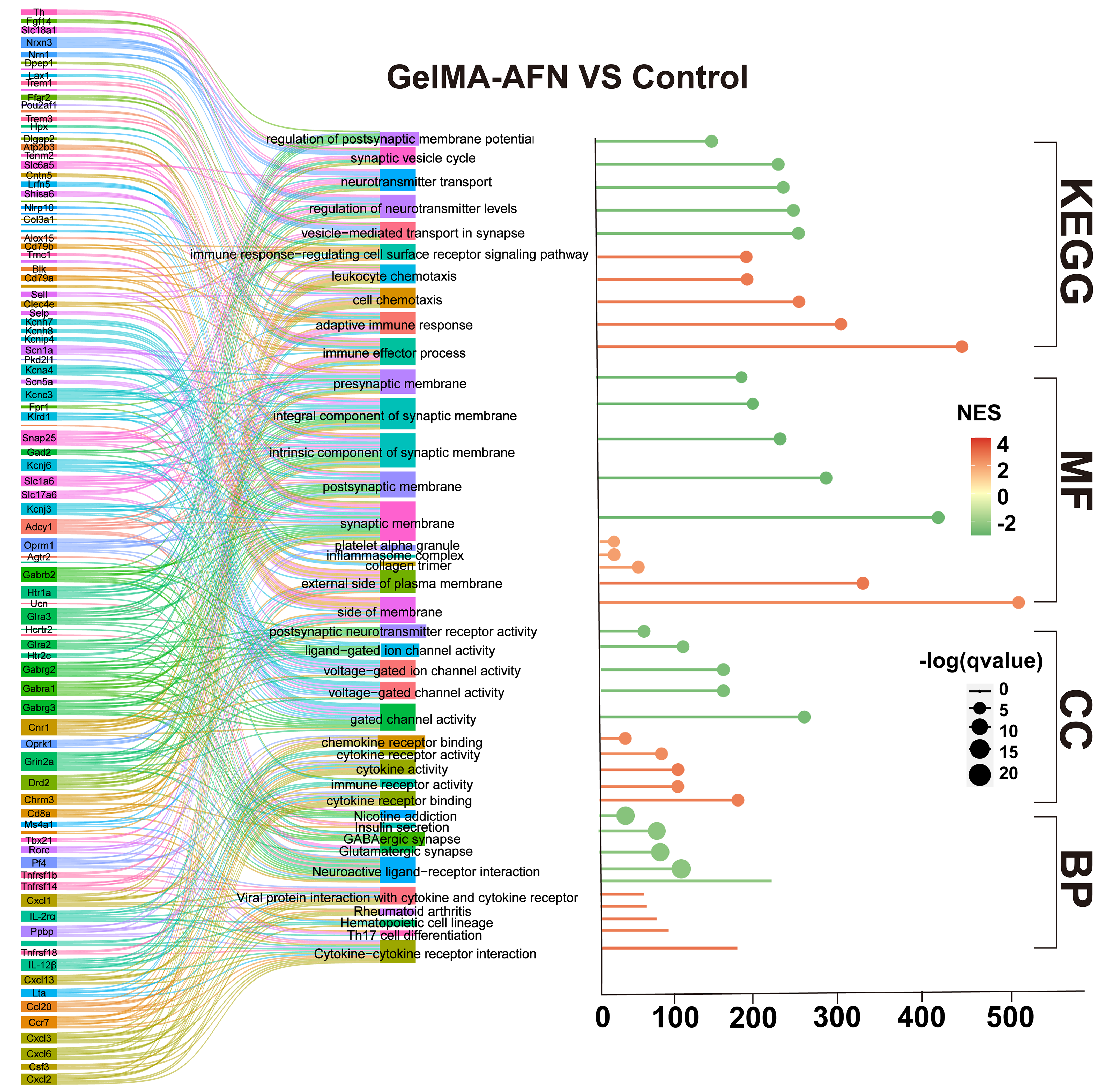

Supplement: Supplementary file 1 — Supplementary figures. [file thnov16p0483s1.zip › Figure S3.tif]
